# Functional changes of the gastric bypass microbiota reactivate thermogenic adipose tissue and systemic glucose control via intestinal FXR-TGR5 crosstalk in diet-induced obesity

**DOI:** 10.1186/s40168-022-01264-5

**Published:** 2022-06-24

**Authors:** Julia Münzker, Nadine Haase, Andreas Till, Robert Sucher, Sven-Bastiaan Haange, Linda Nemetschke, Thorsten Gnad, Elisabeth Jäger, Jiesi Chen, Sjaak J. Riede, Rima Chakaroun, Lucas Massier, Peter Kovacs, Mario Ost, Ulrike Rolle-Kampczyk, Nico Jehmlich, Juliane Weiner, John T. Heiker, Nora Klöting, Gudrun Seeger, Markus Morawski, Verena Keitel, Alexander Pfeifer, Martin von Bergen, Joerg Heeren, Ute Krügel, Wiebke K. Fenske

**Affiliations:** 1 Medical Department III, Endocrinology, Nephrology, Rheumatology, University Hospital of Leipzig, Leipzig, Germany; 2 Department of Internal Medicine I, Division of Endocrinology, Diabetes and Metabolism, University Medical Center Bonn, Bonn, Germany; 3 Department of Visceral-, Transplant-, Thoracic- and Vascular Surgery, University of Leipzig, Leipzig, Germany; 4 Department of Molecular Systems Biology, Helmholtz Centre for Environmental Research Leipzig–UFZ, Leipzig, Germany; 5 Institute of Pharmacology and Toxicology, University Hospital, University of Bonn, Bonn, Germany; 6 Present Address: Department for Pathology, Cedars-Sinai Medical Center Los Angeles, Los Angeles, USA; 7 Department of Neuropathology, University of Leipzig, Leipzig, Germany; 8 German Institute of Human Nutrition Potsdam-Rehbruecke (DIfE), Nuthetal, Germany; 9 Helmholtz Institute for Metabolic, Obesity and Vascular Research (HI-MAG) of the Helmholtz Zentrum München at the University of Leipzig and University Hospital Leipzig, Leipzig, Germany; 10 Paul Flechsig Institute of Brain Research, Faculty of Medicine, University of Leipzig, Leipzig, Germany; 11 Clinic for Gastroenterology, Hepatology and Infectious Diseases, Medical Faculty, University Hospital Düsseldorf, Heinrich-Heine-University, Düsseldorf, Germany; 12 Department of Biochemistry and Molecular Cell Biology, University Medical Center Hamburg-Eppendorf, Hamburg, Germany; 13 Rudolf Boehm Institute of Pharmacology and Toxicology, University of Leipzig, Leipzig, Germany

**Keywords:** Gastric bypass, Gut microbiota, FXR, TGR5, Bile acids, Taurine metabolism

## Abstract

**Background:**

Bariatric surgery remains the most effective therapy for adiposity reduction and remission of type 2 diabetes. Although different bariatric procedures associate with pronounced shifts in the gut microbiota, their functional role in the regulation of energetic and metabolic benefits achieved with the surgery are not clear.

**Methods:**

To evaluate the causal as well as the inherent therapeutic character of the surgery-altered gut microbiome in improved energy and metabolic control in diet-induced obesity, an antibiotic cocktail was used to eliminate the gut microbiota in diet-induced obese rats after gastric bypass surgery, and gastric bypass-shaped gut microbiota was transplanted into obese littermates. Thorough metabolic profiling was combined with omics technologies on samples collected from cecum and plasma to identify adaptions in gut microbiota-host signaling, which control improved energy balance and metabolic profile after surgery.

**Results:**

In this study, we first demonstrate that depletion of the gut microbiota largely reversed the beneficial effects of gastric bypass surgery on negative energy balance and improved glucolipid metabolism. Further, we show that the gastric bypass-shaped gut microbiota reduces adiposity in diet-induced obese recipients by re-activating energy expenditure from metabolic active brown adipose tissue. These beneficial effects were linked to improved glucose homeostasis, lipid control, and improved fatty liver disease. Mechanistically, these effects were triggered by modulation of taurine metabolism by the gastric bypass gut microbiota, fostering an increased abundance of intestinal and circulating taurine-conjugated bile acid species. In turn, these bile acids activated gut-restricted FXR and systemic TGR5 signaling to stimulate adaptive thermogenesis.

**Conclusion:**

Our results establish the role of the gut microbiome in the weight loss and metabolic success of gastric bypass surgery. We here identify a signaling cascade that entails altered bile acid receptor signaling resulting from a collective, hitherto undescribed change in the metabolic activity of a cluster of bacteria, thereby readjusting energy imbalance and metabolic disease in the obese host. These findings strengthen the rationale for microbiota-targeted strategies to improve and refine current therapies of obesity and metabolic syndrome.

Video Abstract

**Graphical abstract:**

Bariatric Surgery (i.e. RYGB) or the repeated fecal microbiota transfer (FMT) from RYGB donors into DIO (diet-induced obesity) animals induces shifts in the intestinal microbiome, an effect that can be impaired by oral application of antibiotics (ABx). Our current study shows that RYGB-dependent alterations in the intestinal microbiome result in an increase in the luminal and systemic pool of Taurine-conjugated Bile acids (TCBAs) by various cellular mechanisms acting in the intestine and the liver. TCBAs induce signaling via two different receptors, farnesoid X receptor (FXR, specifically in the intestines) and the G-protein-coupled bile acid receptor TGR5 (systemically), finally resulting in metabolic improvement and advanced weight management. BSH, bile salt hydrolase; BAT brown adipose tissue.
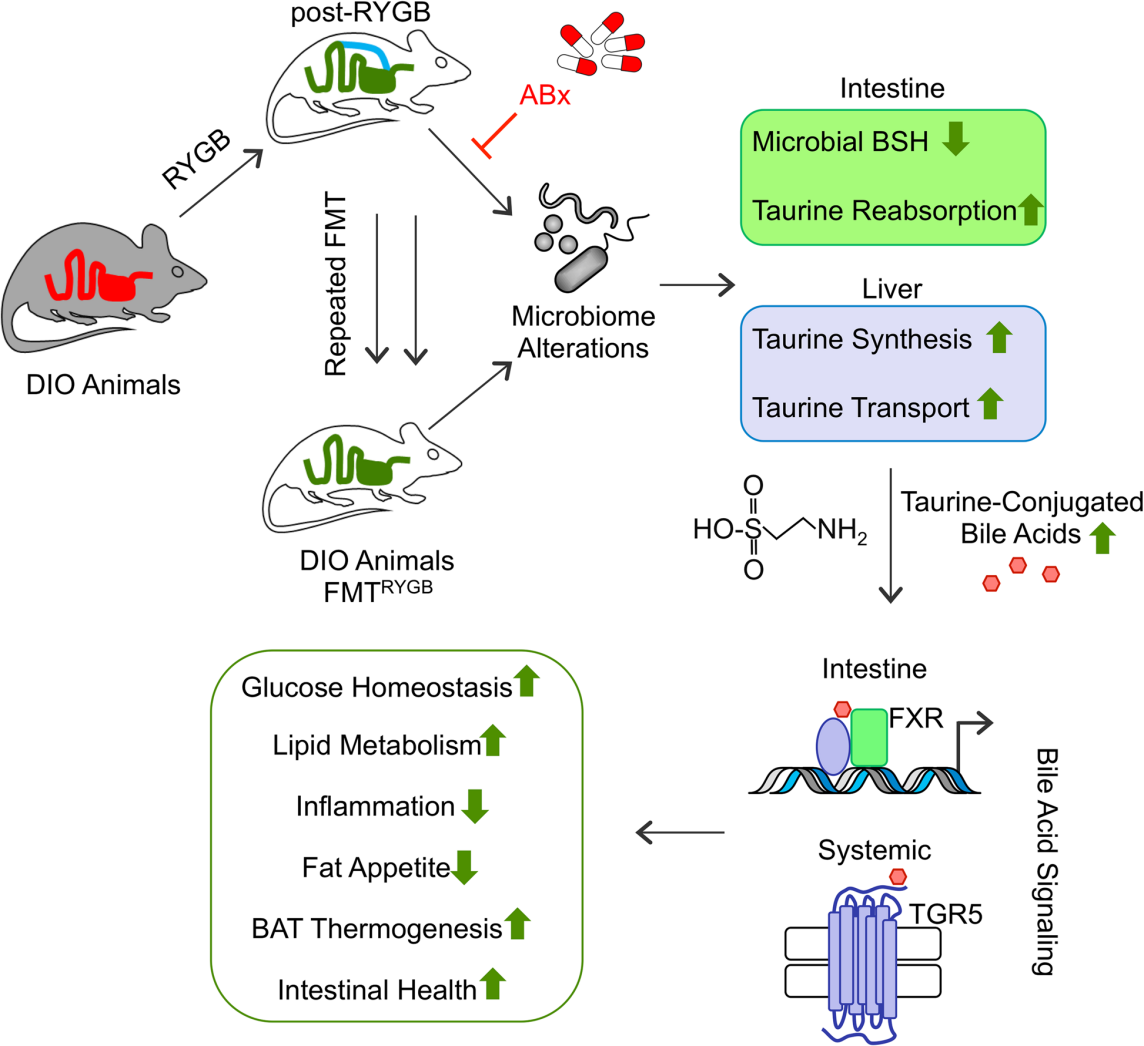

**Supplementary Information:**

The online version contains supplementary material available at 10.1186/s40168-022-01264-5.

## Background

Bariatric surgery remains the most effective treatment for obesity and related comorbidities [1, 2]. However, its invasive and irreversible character limits surgery for a minority of patients in need of therapy. Efforts to uncover mechanistic concepts of surgical intervention into efficient and less invasive anti-obesity strategies constitute a major challenge to medical research today. As the prime target of surgery, the altered intestinal architecture and particularly the metabolic milieu of its bacteria present a promising mechanistic starting point. Accumulating evidence has linked the gut microbiome to human obesity and metabolic disorders, which associate with reduced microbial diversity and gene richness [3–6]. Interestingly, pronounced changes in the gut microbiota composition were demonstrated after Roux-en-Y gastric bypass (RYGB) surgery in obese humans [7–12] and rodents [13–15], and associations have been reported between taxonomic shifts and metabolic outcome after bariatric surgery in humans [9, 12, 16] and rats [17]. Moreover, recent proof-of-principle studies on fecal transplantation from RYGB operated humans [11] and mice [13] into germ-free animals suggest that the gut microbiota may control reduced post-RYGB fat storage.

However, the essential question as to the mechanistic basis of the altered gut microbiota as an environmental regulator of improved energy homeostasis and metabolism after RYGB remains unaddressed. Moreover, evidence for a possibly inherent therapeutic concept of post-surgery modulated bacterial metabolic activity against obesity and metabolic syndrome is missing.

Here, we systematically dissected the complex relation between the post-RYGB altered gut microbiome and reinstatement of host metabolic health in high-fat diet-induced obesity (HF-DIO). Firstly, by depleting the gut microbiota in rats subjected to RYGB surgery, we investigated the principal role of the gut microbiota in surgery-induced weight loss and improved metabolic control. Secondly, we examined via fecal transplant experiments whether the isolated RYGB gut microbiota is capable to improve adiposity and related metabolic derangements in non-operated HF-DIO recipients. Finally, we integrated 16S rRNA sequencing, metabolomics, and metaproteomics to characterize the functional signature of the gut microbiota in divergent metabolic traits and to identify microbiota-regulated molecular targets, which beneficially integrate the RYGB gut microbiota to host energetics and metabolic health.

## Results

### Microbiota depletion impairs weight loss and metabolic improvements resulting from RYGB surgery in diet-induced obesity

**Fig. 1:**
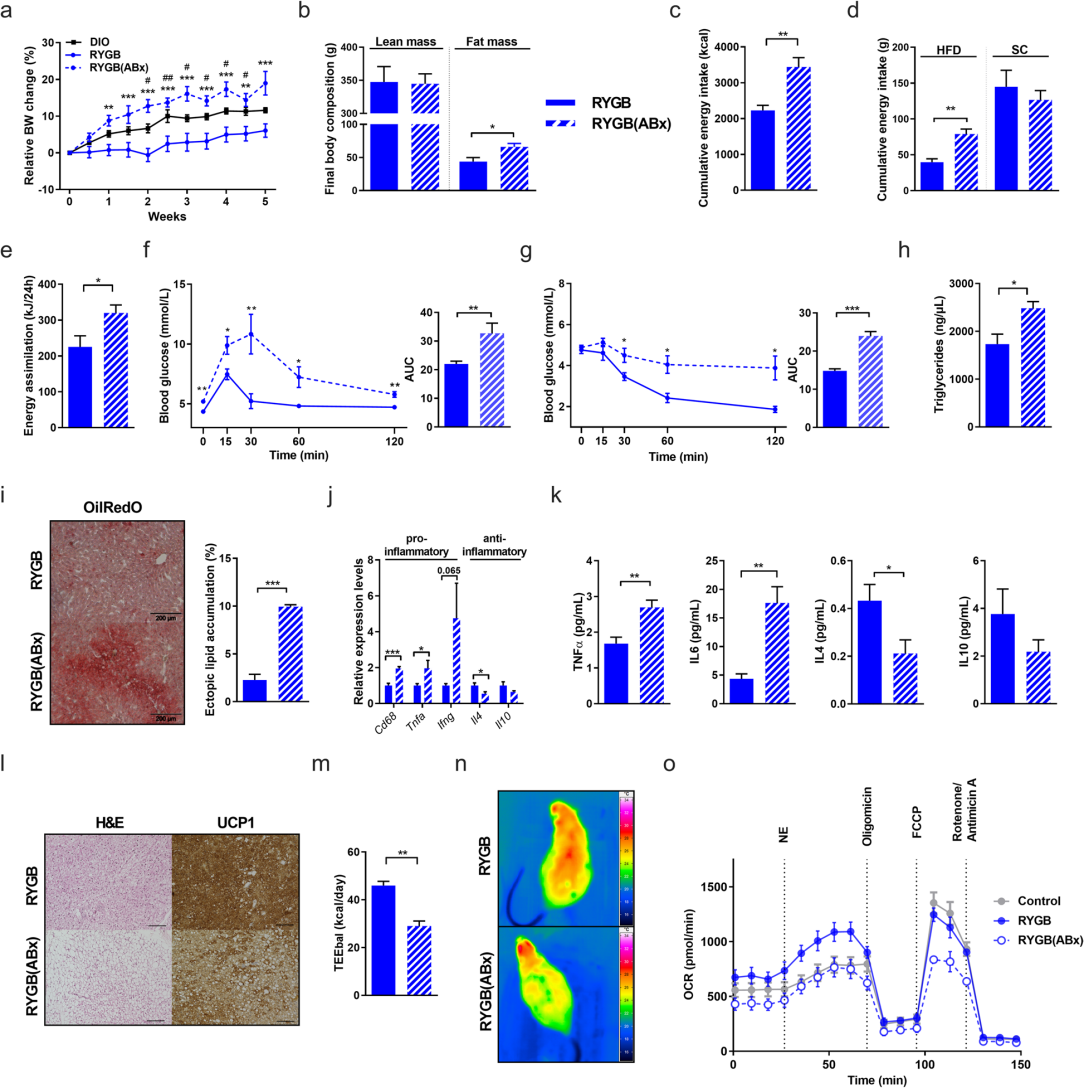
Microbiota depletion impairs weight loss and metabolic improvements resulting from RYGB surgery in diet-induced obesity. **a**–**f** Relative body weight change (in %; average body weight (øBW) per group: RYGB(ABx) week (W)0: 385.6 g, W5: 454.3 g; DIO W0: 681.3 g, W5: 762.3 g; RYGB W0: 415.3 g, W5: 437.7 g; # DIO vs. RYGB; * RYGB vs. RYGB(ABx)) (**a**), final body composition with lean and fat mass (in g), **b** cumulative energy intake (in kcal) (**c**), cumulative energy intake of high fat diet (HFD) and standard chow (SC) (in g) (**d**), energy assimilation (in kJ/24 h) (**e**), oral glucose-tolerance tests (oGTT) (**f**), and insulin-sensitivity assessed by insulin-tolerance tests (ITT) (**g**) in RYGB-operated rats with 35-day antibiotic-treatment (ABx) compared to their respective controls (RYGB-operated rats without ABx). **h**–**k** Fasting plasma triglyceride concentrations (in ng/μl) (**h**), representative images (at least 4 images per group) of liver Oil Red O staining (scale bars 200 μm) with quantification of hepatic lipid accumulation (in %) (**i**), and relative mRNA expression of hepatic pro- and anti-inflammatory cytokines (**j**) in RYGB(ABx) compared to RYGB rats. Plasma cytokine concentrations in RYGB(ABx) and RYGB rats (in pg/ml) (**k**). **l-n** Representative images of H&E and UCP1 staining in BAT (scale bars 200 μm) (**l**), total energy expenditure (TEE; in kcal/day) (**m**) and infrared images of representative RYGB(ABx) or RYGB rats after 6 h of cold-exposure (**n**). Mouse primary brown adipocytes were treated for 6 h with 50% (vol/vol) of rat serum from RYGB(ABx) or RYGB rats (or with PBS as control). Oxygen consumption rate was measured on a respirometry Seahorse XF Cell Mito Stress Test (Agilent) as described in the “Methods” section (**o**). Experiments were performed in three independent cohorts. Data are mean ± s.e.m; *n* = 3–8 animals per group with pooled data from 2 to 3 independent experiments. *,#*P* < 0.05, **,## *P* < 0.01, ***,### *P* <0.001 as assessed by unpaired Student’s *t* test (for two groups) or two-way ANOVA (multiple groups) with Tukey correction for multiple testing

To study the role of the persistent post-RYGB microbial signature [13] in postoperative weight loss maintenance and improved systemic metabolism, we established RYGB surgery in conventional raised HF-DIO rats [18, 19] and treated animals at a stage of sustained weight reduction with oral broad-spectrum antibiotics (ABx) for conditional microbiota depletion (RYGB(ABx)) [20, 21]. Postoperatively, animals were switched to a two-choice diet between HFD and SC, resembling the free nutrient choice situation under real-life conditions (Supplementary Figure S1). The same antibiotic regimen as received by the RYGB group was also administered to a standard chow (SC)-fed group to control for specificity and potential pharmacological side effects (Supplementary Figure S2). Antibiotic treatment caused a reduction of fecal bacterial load by approximately factor 10 as assessed by bacterial DNA-specific quantitative real-time PCR and normalization of DNA detection levels to fecal mass (Supplementary Figure S3a). Notably, we found that ABx-induced microbiota depletion significantly reduced the beneficial effects of RYGB on reduced adiposity. While the RYGB group exhibited a significantly lower weight gain than sham-operated controls under the same diet regimen (Fig. 1a), RYGB animals under antibiotic treatment (RYGB(ABx)) revealed a more than 3-fold higher weight gain than RYGB littermates within 5 weeks. Increased body weight mainly resulted from fat mass gain (Fig. 1b) and was linked to increased cumulative energy intake (Fig. 1c), predominantly resulting from a switch to high-fat diet preference (Fig. 1d, Supplementary Figure S3b), and to increased energy assimilation (Fig. 1e).

Interestingly, RYGB-induced benefits on body weight relapsed shortly after onset of antibiotics and compromised metabolic health as demonstrated by reduced glucose clearance (Fig. 1f), compromised insulin sensitivity (Fig. 1g), elevated blood triglycerides (Fig. 1h) together with histological features of hepatic steatosis (Fig. 1i, j), and a systemically dominant pro-inflammatory cytokine profile (Fig. 1k) after 5 weeks of treatment. These systemic metabolic derangements were not linked to changes in fecal energy loss or lipid levels in fecal content (as determined for sphingomyelins, acylcarnitines, and phosphatidylcholines, *data not shown*). Importantly, ABx had no effect on body weight (Supplementary Figure S2a), cumulative energy intake, food efficiency, energy excretion, and assimilation (Supplementary Figure S2d, e) nor on glucose control (Supplementary Figure S2f, g) or brown adipose tissue mass (Supplementary Figure S2h) in SC-fed controls.

Given the pivotal role of the gut microbiome in processing and absorption of nutrients for subsequent energy provision to thermogenic fat tissues, we next examined in more detail the relevance of the RYGB microbiome for energy metabolism in thermogenic adipose tissue. Consistent with adiposity and prominent morphological changes of both white adipose tissue (WAT) depots (*data not shown*), microbial depletion in RYGB(ABx) led to considerable remodeling of the interscapular brown adipose tissue (iBAT), as histologically characterized by substantial whitening of iBAT with prominent accumulation of lipid droplets and reduced protein expression of the BAT marker uncoupling protein 1 (UCP1) in immunostaining (Fig. 1l). In addition, microbiota depleted animals showed significantly reduced energy expenditure (Fig. 1m) and lower body temperature during cold exposure compared to RYGB littermates (Fig. 1n), indicating impaired adaptive thermogenesis following microbiota depletion. We therefore hypothesized that bacteria-derived circulating signals might mediate a crosstalk between gut microbiota and thermogenically active BAT depot. To address this hypothesis, we measured mitochondrial respiration in brown adipocytes, obtained from the stromal vascular fraction of mouse BAT as previously described [22], after cell pre-treatment with serum derived from RYGB or RYGB(ABx) animals. Interestingly, while pretreatment with serum from RYGB animals significantly increased basal as well as maximal respiration in brown adipocytes compared to PBS as control, pretreatment with serum from RYGB(ABx) animals significantly blunted basal as well as maximal mitochondrial respiration (Fig. 1o, Supplementary Figure S3c–e). Moreover, mitochondrial oxygen consumption significantly increased after norepinephrine stimulation of RYGB serum pre-treated brown adipocytes, whereas pre-treatment with RYGB(ABx) impaired this stimulatory effect (Supplementary Figure S3e). Taken together, these microbiota depletion experiments in RYGB provide first evidence that the microbiota modulation in RYGB is an essential mediator of weight loss and metabolic benefits after gastric bypass surgery and critically linked to reduced fat appetite and stimulated BAT thermogenic activity.

### RYGB gut microbiota transfer counters adiposity and metabolic disease in HFD-induced obesity

Having shown that the post-RYGB microbiome is *necessary* for weight loss maintenance and metabolic improvements upon surgery, we next investigated whether it is also *sufficient* to improve adiposity and related metabolic disorders through transfer in a model of diet-induced obesity. Importantly, and as a critical distinction to previous experiments on germ-free mice [11, 13], which are protected from diet-induced obesity and related metabolic disease [4, 23, 24], we here chose a conventionally raised disease model and inoculated HF-DIO rats harboring an obesogenic microbiome with fresh fecal microbiota isolated from RYGB-operated donors (Supplementary Figure S1).

To control for donor specificity, another group of HF-DIO rats was inoculated with fresh fecal microbiota from SC-fed lean donors (Supplementary Figure S4).

**Fig. 2:**
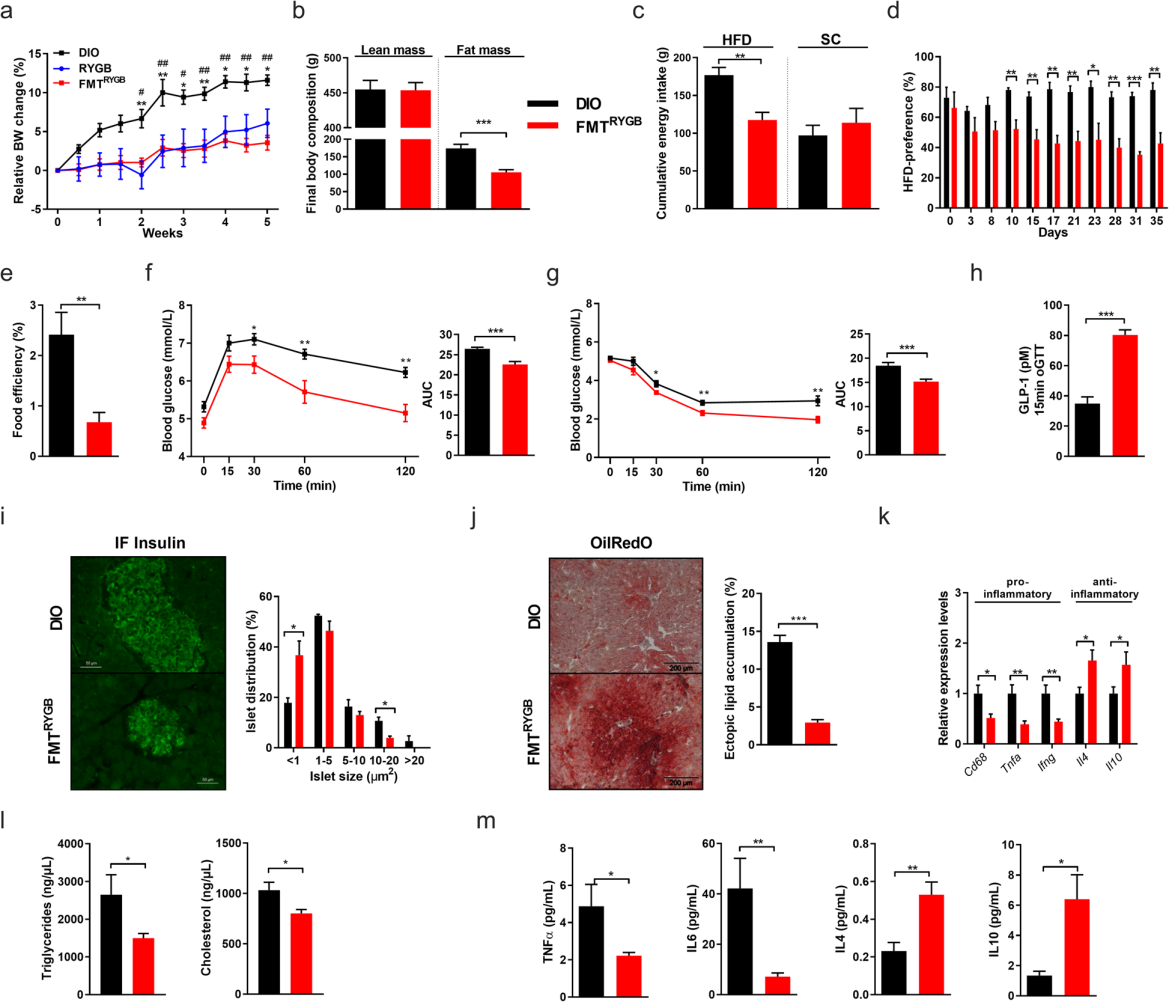
RYGB gut microbiota transfer counters adiposity and metabolic disease in conventionally raised HFD-induced obesity. **a**–**e** Relative body weight change (in %; øBW per group: DIO W0: 681.3 g, W5: 762.3 g; FMT^RYGB^ W0: 528 g, W5: 595.2 g; RYGB W0: 415.3 g, W5: 437.7 g; * DIO vs. RYGB; # DIO vs. FMT^RYGB^) (**a**), final body composition with lean and fat mass (in g) (**b**), cumulative energy intake of HFD and SC (in g) (**c**), HFD preference (in %) (**d**) and food efficiency (in %) (**e**) in HFD-induced obese (DIO) rats 5 weeks after fecal microbiota transfer (FMT) from RYGB-operated rats (FMT^RYGB^) compared to DIO controls. **f**–**i** Oral glucose-tolerance tests (oGTT) (**f**), insulin sensitivity assessed by insulin-tolerance tests (ITT) (**g**), and plasma GLP-1 (in pM) release at 15 min after oral glucose exposure (**h**) 5 weeks after FMT. Representative immunofluorescent (IF) insulin-stained pancreas sections (scale bars 50 μm) and quantification of pancreatic islet sizes (in %) in FMT^RYGB^ rats and DIO controls (**i**). **j**–**m** Liver OilRedO staining (scale bars 200 μm) with quantification of hepatic lipid accumulation (in %) (**j**). Hepatic cytokine gene expression (**k**), plasma lipid (in ng/μl) **(l),** and plasma cytokine levels (in pg/ml) (**m**) in FMT^RYGB^ rats and DIO controls. Note that for clarity purposes and in order to reduce animal numbers, data from groups DIO and FMT^RYGB^ have exceptionally also been used in Supplementary Figure S5. Data are mean ± s.e.m; *n* = 3–8 animals per group with pooled data from 2 to 3 independent experiments. *,# *P* < 0.05, **,## *P* < 0.01, ***,### *P* < 0.001 by unpaired Student’s *t* test (for two groups) or two-way ANOVA (multiple groups) with Tukey correction for multiple testing

Notably, HF-DIO recipients colonized with microbiota from RYGB-treated donors (FMT^RYGB^) largely mimicked the weight curve and feeding behavior of their microbial donors [18, 19], as shown by a significant decrease in weight gain and adiposity (Fig. 2a, b) and a clear loss in fat appetite (Fig. 2c, d), resulting in reduced cumulative energy intake. Interestingly, despite no difference in energy digestibility (*not shown*), rats colonized with RYGB microbiota showed reduced ability to convert dietary energy to body mass (Fig. 2e), indicating an increased metabolic efficiency compared to HF-DIO.

Pursuing on this, we found that obese animals transplanted with RYGB microbiota (other than with lean microbiota, Supplementary Figure S3), mimic major aspects of surgical RYGB reconfiguration on energy dissipation and metabolic health benefits. FMT^RYGB^ rats showed improved glucose tolerance (Fig. 2f), peripheral insulin sensitivity (Fig. 2g), and reduced hepatic glucose production (*not shown*), arguing for enhanced liver insulin sensitivity. Moreover, improved glucose control was linked to an increase in GLP-1 release in response to oral glucose load (Fig. 2h) and less hypertrophic pancreatic islets in immunofluorescent staining compared to HF-DIO controls (Fig. 2i).

In line with reduced adiposity and improved insulin sensitivity, FMT^RYGB^ animals showed improved steatosis (Fig. 2j–k), reduction in lipid levels (Fig. 2l), and a switch to a dominant anti-inflammatory cytokine profile compared to HF-DIO controls (Fig. 2m).

Importantly, energetic and metabolic benefits following RYGB microbiota transfer could only partly be attributed to lower caloric intake, as pair feeding experiments (matched to the composition and amount of food consumed by the FMT^RYGB^ group) entailed clearly less pronounced adiposity reduction and metabolic benefits than in the FMT^RYGB^ group (Supplementary Figure S5). Together these data demonstrate that the transfer of the RYGB microbiota is *sufficient* to exert major therapeutic aspects of surgical intervention on energy balance and metabolism in our rodent model of HF-DIO.

### RYGB microbiota transfer reduces inflammation and enhances lipolysis and energy expenditure from brown adipose tissue in HFD-induced obesity

As the beneficial effects of RYGB microbiota transfer on host energetics were only partly attributable to feeding suppression and fecal energy loss, we further focused on the postulated link between RYGB microbiota transfer and downstream signaling responses to regulate adaptive thermogenesis in brown adipocytes. Interestingly, recent studies in mice reported that the browning process of WAT [25, 26] and the metabolic activity of BAT [27] is responsive to the gut microbiome and its environmental modulation. Still, little is known about the regulatory mechanisms how bacterial downstream signaling processes may control or even activate energy dissipation in obesity and related diseases.

**Fig. 3:**
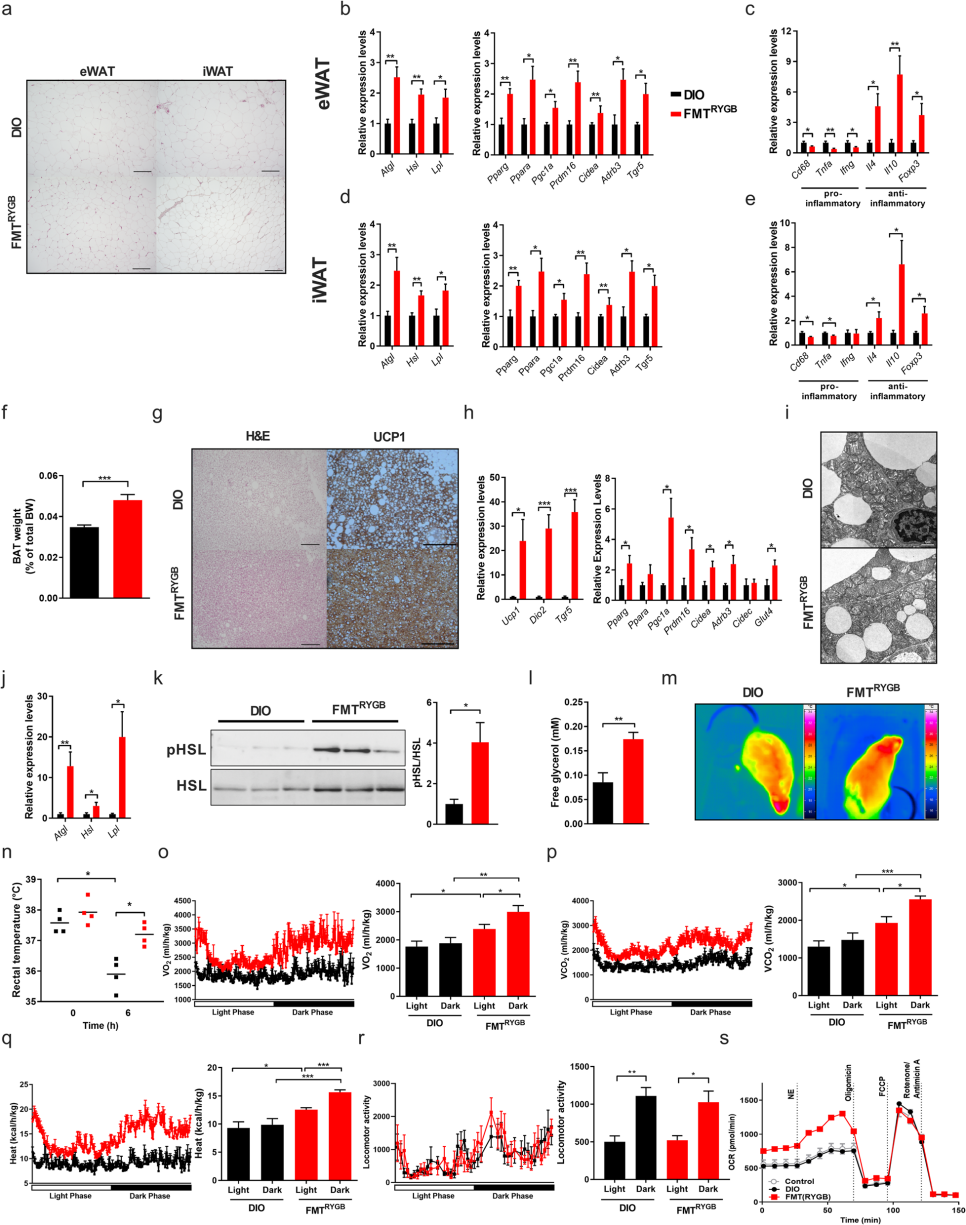
RYGB microbiota transfer reduces inflammation and enhances lipolysis and thermogenic activity in adipose tissue. **a**–**e** H&E staining on sections from epididymal (eWAT) and inguinal (iWAT) white adipose tissue (scale bars 200 μm) 5 weeks after fecal microbiota transfer (FMT) from RYGB-operated rats (FMT^RYGB^) compared to their respective controls (DIO rats not receiving RYGB microbiota) (**a**). Relative mRNA expression of genes involved in lipolysis, fatty acid oxidation, and inflammatory cytokines in eWAT (**b**, **c**) and iWAT (**d**, **e**). **f**–**i** BAT weight (in % of total BW) (**f**), H&E and UCP1 staining of BAT sections (scale bars 200 μm) (**g**) and relative mRNA expression of genes involved in fatty acid oxidation and thermogenesis in BAT (**h**). Electron microscopy of ultra-thin sections of BAT depicting mitochondrial fine structure in DIO vs FMT^RYGB^ animals (scale bar 1 μm) (**i**). **j**–**l** Relative mRNA expression of genes involved in lipolysis in BAT (**j**), phosphorylation levels of HSL (p-HSL) **(k)** and free plasma glycerol levels (in mM) (**l**). **m**–**r** Representative infrared images (**m**) and corresponding rectal body temperature (in °C) after 6 h of cold-exposure (**n**). Oxygen consumption (VO_2_; in ml/(h*kg)) (**o**), carbon dioxide production (VCO_2_; in ml/(h*kg)) (**p**), heat production (in kcal/(h*kg)) (**q**) and locomotor activity (**r**) of FMT^RYGB^ and DIO rats (at 5 weeks of FMT treatment). Seahorse analysis of murine adipocytes after pretreatment with rat serum (DIO vs. FMT^RYGB^, PBS as control) (**s**). Data are mean ± s.e.m; *n* = 3–8 animals per group with pooled data from 2 to 3 independent experiments. *P < 0.05, ** *P* < 0.01, *** *P* < 0.001; unpaired Student’s *t* test

We first examined the modulating effect of RYGB microbiota transfer on WAT and found here clearly reduced adipocyte cell sizes (Fig. 3a, Supplementary Figure S6a, b) and increased expression of target genes involved in lipolysis and thermogenesis in epididymal and inguinal WAT (eWAT, iWAT, respectively) of FMT^RYGB^ compared to DIO (Fig. 3b, d).

Moreover, and consistent with reduced adiposity and systemic metaflammation in FMT^RYGB^ (Fig. 2k, m), a shift towards a dominant anti-inflammatory state was found in the WAT depots (Fig. 3c, e) together with reduced extracellular matrix remodeling and tissue fibrosis (Supplementary Figure S6c, d), both pathological conditions strongly linked to insulin resistance and metabolic dysfunction [28].

Beyond profound WAT remodeling, a major recruitment of iBAT mass was found in FMT^RYGB^ animals (Fig. 3f), enriched with UCP1-positive cells and depleted in lipid content (Fig. 3g). The enlarged tissue mass showed increased thermogenic gene expression (Fig. 3h), a higher number of lamellar cristae in the enlarged mitochondria (Fig. 3i), and substantially reduced tissue fibrosis as compared to HF-DIO controls (Supplementary Figure S6e).

As BAT thermogenesis is fueled by mitochondrial oxidation of free fatty acids [29, 30], we quantified lipolytic cleavage of triglycerides and found increased expression of lipolytic genes (Fig. 3j) and higher activity of hormone sensitive lipase (HSL) (Fig. 3k) in iBAT. Consequently, we measured elevated plasma glycerol levels (Fig. 3l) in FMT^RYGB^ rats compared to HF-DIO controls.

Consistent with an activation in thermogenesis, at functional level in vivo, FMT^RYGB^ animals showed an improved cold tolerance (Fig. 3m, n) and higher levels of 24h O_2_ consumption, CO_2_, and heat production compared to HF-DIO. These observations were complemented by strong amplitudes of circadian oscillations re-entrained by light-dark cycles in FMT^RYGB^ as measured by indirect calorimetry tracings (Fig. 3o–q). Differences in heat production did not derive from differences in locomotor activity (Fig. 3r), together arguing for higher mobilization of energy for an accelerated metabolic rate.

Interestingly, and in contrast to our findings in the microbiota-depleted RYGB(ABx) group (Fig. 1o; Supplementary Figure S3c–e), treatment of murine brown adipocytes with serum from FMT^RYGB^ recipients elicits a substantial increase in basal, norepinephrine-stimulated and maximal mitochondrial respiration compared to control or treatment with serum from HF-DIO (Fig. 3s; Supplementary Figure S6f–h).

Together, these data provide strong evidence that the RGYB microbiome, presumably through downstream signaling factors in the circulation derived from altered microbial metabolic activity, is able to stimulate energy dissipation and metabolic rate in HF-DIO by accelerating BAT activity. Moreover, these findings suggest a mechanistic explanation for improved glucose and lipid metabolism beyond feeding suppression, where the RYGB microbiome orchestrates a coordinated metabolic response that promotes lipolysis and mobilizes free fatty acids (FFAs) from plasma triglycerides for increased thermogenic activity.

### RYGB microbiota transfer modulates taurine metabolism and intestinal BA receptor signaling linked to improved intestinal health in HFD-induced obesity

Having demonstrated a causal interplay between RYGB microbiota and host energy and metabolic balance, we sought to gain more mechanistic insight into the functional gut microbiota-host interaction jointly directing weight loss and metabolic reprogramming in the obese host organism.

We therefore profiled the cecal microbial composition from metabolically healthy donors and recovered recipients of RYGB microbiota as well as from metabolically compromised HF-DIO and RYGB(ABx) animals, and searched for corresponding surgery-triggered and microbiota-transmitted taxonomic changes of the gut microbial composition by 16S rRNA gene sequencing.

**Fig. 4:**
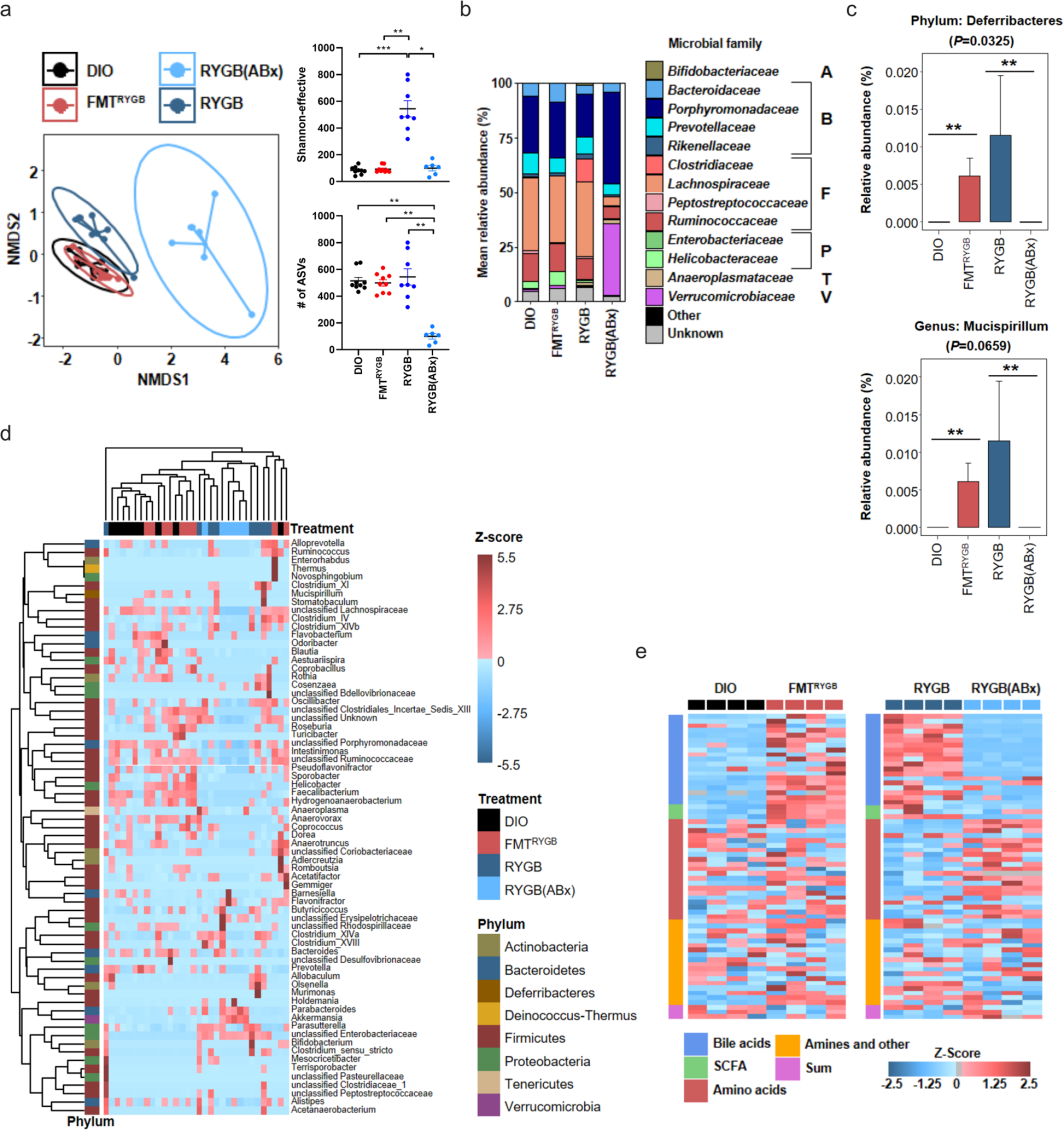
Modulation of the gut microbiome by RYGB surgery, RYGB microbiota transfer (FMT^RYGB^) and RYGB microbiota deletion (RYGB(ABx)) in HFD-induced obesity (DIO). **a**–**c** Non-metric multidimensional scaling (NMDS) dissimilarity analysis of 16S rRNA gene profiling data, alpha diversity, and richness based on amplicon sequence variant (ASV)-count (**a**). Mean relative abundance of bacterial families and phyla (A = *Actinobacteria*, B = *Bacteroidetes*, F = *Firmicutes*, P = *Proteobacteria*, T = *Tenericutes*, V = *Verrumicrobiota*) (**b**). Significantly altered Taxa (**c**). Distribution and hierarchical clustering of microbial genera (**d**). Heatmap of portal vein plasma metabolites of respective groups (**e**). *n* = 6–10 animals per group with pooled data from 3 independent experiments; *P < 0.05, ** *P* < 0.01, *** *P* < 0.001

Beta-diversity analysis demonstrated that antibiotic treatment altered the RYGB microbial signature as expected, while other bacterial profiles (HF-DIO and FMT^RYGB^) revealed substantial compositional overlap as reported by non-metric multidimensional scaling (NMDS) dissimilarity analysis (Fig. 4a). This finding is in line with other preclinical [31] and clinical [16] FMT studies using conventionalized recipients. Notably, the reduced alpha-diversity in HF-DIO, based on the Shannon-effective score, did not recover with metabolic improvement following RYGB surgery or microbiota transfer (Fig. 4a). Analyzing the bacterial composition at different levels, we found strongly perturbed bacterial taxa in the RGYB(ABx) group (Fig. 4b), whereas FMT^RYGB^ animals specifically enriched *Deferribacteres* as compared to HF-DIO controls (Fig. 4c, d). This difference could be traced to enriched abundance at the genus level (Fig. 4c), while major parts of the HF-DIO taxonomic composition persisted in FMT^RYGB^. These data implicate that less specific bacteria, but rather a collective change in metabolic activity in a cluster of bacteria may direct metabolite downstream signaling, which orchestrates beneficial energy and metabolic balance in HF-DIO following RYGB microbiota transfer.

As microbial metabolites are pivotal mediators of host-microbiota communication [32], we hypothesized that modulated metabolite signaling may contribute to improved metabolic outcome upon RYGB surgery and RYGB microbiota transfer. To this end, we performed a metabolomics screen in portal vein plasma (Fig. 4e) and caecum (Supplementary Figure S7) to focus on the most differentially abundant metabolites, whose levels were depleted in HF-DIO and RYGB(ABx) rats or rather regained upon RYGB surgery and RYGB microbiota transfer (FMT^RYGB^).

**Fig. 5:**
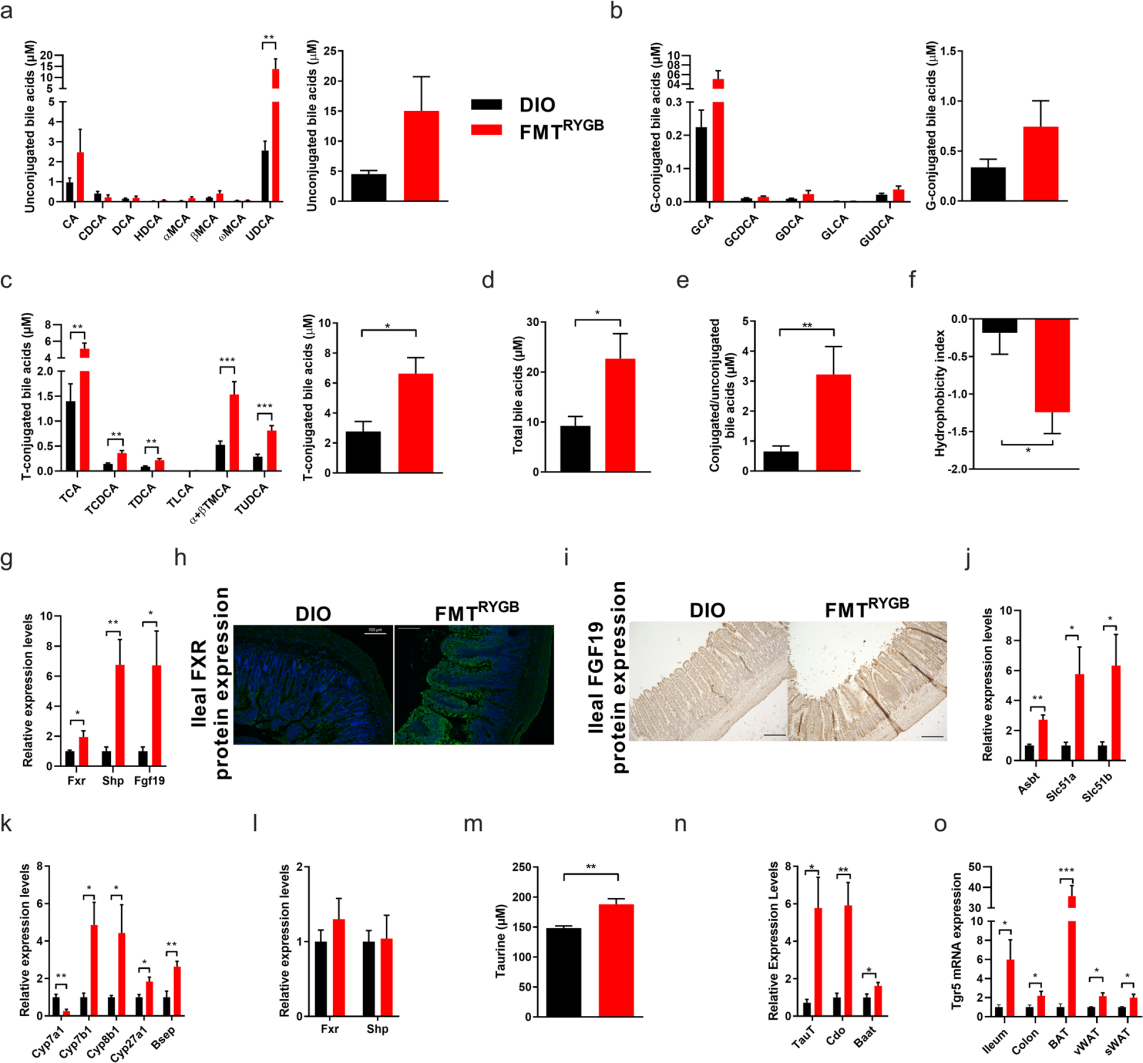
RYGB microbiota transfer modulates intestinal BA receptor signaling and promotes intestinal health in HFD-induced obesity. **a**–**e** Concentrations of unconjugated BA (UBA) species (in μM) (**a**), glycine (G)- (**b**) and taurine (T)-conjugated BA (CBA) species (in μM) (**c**), and of total bile acids in plasma (in μM) (**d**), ratio of total CBA to UBA (**e**) and hydrophobicity index (**f**) in plasma from portal vein collected at 5 weeks after fecal microbiota transfer (FMT) from RYGB-operated rats (FMT^RYGB^) compared to their respective controls (DIO rats not receiving RYGB microbiota). **g–j** Relative mRNA expression of FXR target genes in ileum (**g**), representative immunofluorescent FXR-stained ileal sections (scale bars 100 μm) (**h**) and immunohistochemistry FGF19-stained ileal sections (scale bars 200 μm) (**i**), and relative mRNA expression of target genes involved in BA transport in the ileum (**j**) of FMT^RYGB^ and DIO animals. **k**, **l** Hepatic mRNA expression of genes involved in BA metabolism (**k**) and FXR target genes (**l**). **m**–**o** Plasma free taurine concentrations (**m**) and relative mRNA expression of hepatic target genes involved in taurine metabolism (**n**). Relative mRNA expression (normalized to DIO) of *Tgr5* in ileum and additional organs (**o**). Data are mean ± s.e.m; *n* = 6–10 animals per group with pooled data from 3 independent experiments. *P < 0.05, **P < 0.01, ***P < 0.001; unpaired two-tailed Student’s *t* test

Among these metabolites were several bile acids (BA), which is a critical class of metabolic regulators that is involved in glucose, lipid and energy control mainly *via* the nuclear farnesoid X receptor (FXR) and the transmembrane G protein-coupled receptor 5 (TGR5) [33]. While several studies previously linked altered BA signaling with metabolic health benefits upon bariatric surgery [11, 34–36], the mechanistic basis of post-surgery altered BA signaling and its functional transferability by transplantation into colonized HF-DIO remains unknown. Here, we hypothesized that persistent and transferable shifts in the metabolic activity of bacteria clusters upon RYGB may redirect the manifest BA dysmetabolism in transplanted DIO [37] towards enhanced BA substrate synthesis and selective receptor signaling. In line with this hypothesis, we found increased levels of total and taurine-conjugated BA (T-CBA) pool sizes in portal plasma (Fig. 5a–d) and the caecum (Supplementary Figure S7) in FMT^RYGB^ animals. Moreover, circulating levels of cytotoxic BAs (as assessed by hydrophobicity index, Fig. 5f) were reduced in FMT^RYGB^ together with an activated ileal FXR-FGF19 axis (Fig. 5g–i) and increased expression of genes involved in ileal BA re-uptake as part of the enterohepatic circulation (Fig. 5j).

Moreover, shifts in the portal plasma BA composition in FMT^RYGB^ were associated with a switch from the classical to the alternative BA biosynthesis pathway in the liver (Fig. 5k), which has previously been reported as a key factor for BAT recruitment upon cold challenge [38, 39] and the beiging process in WAT upon intestinal FXR activation in mice [40].

Interestingly, mRNA expression levels of hepatic *Fxr* and its downstream target small heterodimer partner (*Shp)* were unaltered (Fig. 5l), indicating absent activation of hepatic FXR upon RYGB microbiota transfer.

To identify the basis of the prominent induction of plasma T-CBA in FMT^RYGB^, we quantified free taurine by targeted mass spectrometry and found increased levels in portal plasma (Fig. 5m) and cecal lumen (Supplementary Figure S7). Consistent with that, expression of hepatic genes involved in taurine biosynthesis and transport (Fig. 5n) were enhanced in FMT^RYGB^ recipients. These data suggest that selective induction of the alternative pathway increases BA pool size coupled to taurine, and that saturation of the intestinal BA transporters may promote increased abundance of cecal T-CBA concentrations following RYGB microbiota transfer compared to HF-DIO.

Moreover, increased systemic T-CBA pool size and intestinal FXR signaling was linked to enhanced intestinal and systemic *Tgr5* expression in FMT^RYGB^ (Fig. 5o). Together with an increase in GLP-1 release (Fig. 2h) and ileal expression of proprotein convertase 1 (*Pcsk1*) and *Pyy* (Supplementary Figure S8), these findings may argue for mechanistic involvement of the recently discovered intestinal BA receptor crosstalk [40–42], where activation of intestinal *Fxr* shapes the microbiota to induce *Tgr5*-mediated glucose control [41, 42] and BAT activity via cAMP-dependent signaling [37]. Importantly, increased T-CBA pool size showed no signs of intestinal toxicity (Supplementary Figure S8). In contrast, FMT^RYGB^ recipients revealed strong morphological and functional signatures of improved intestinal health and barrier function compared to HF-DIO (Supplementary Figure S8), which is consistent with previously reported effects of intestinal FXR activation [40, 42]. Taken together, these findings indicate that the RYGB microbiome harbors strong entero-protective properties in HF-DIO being transmissible via fecal transfer.

**Fig. 6:**
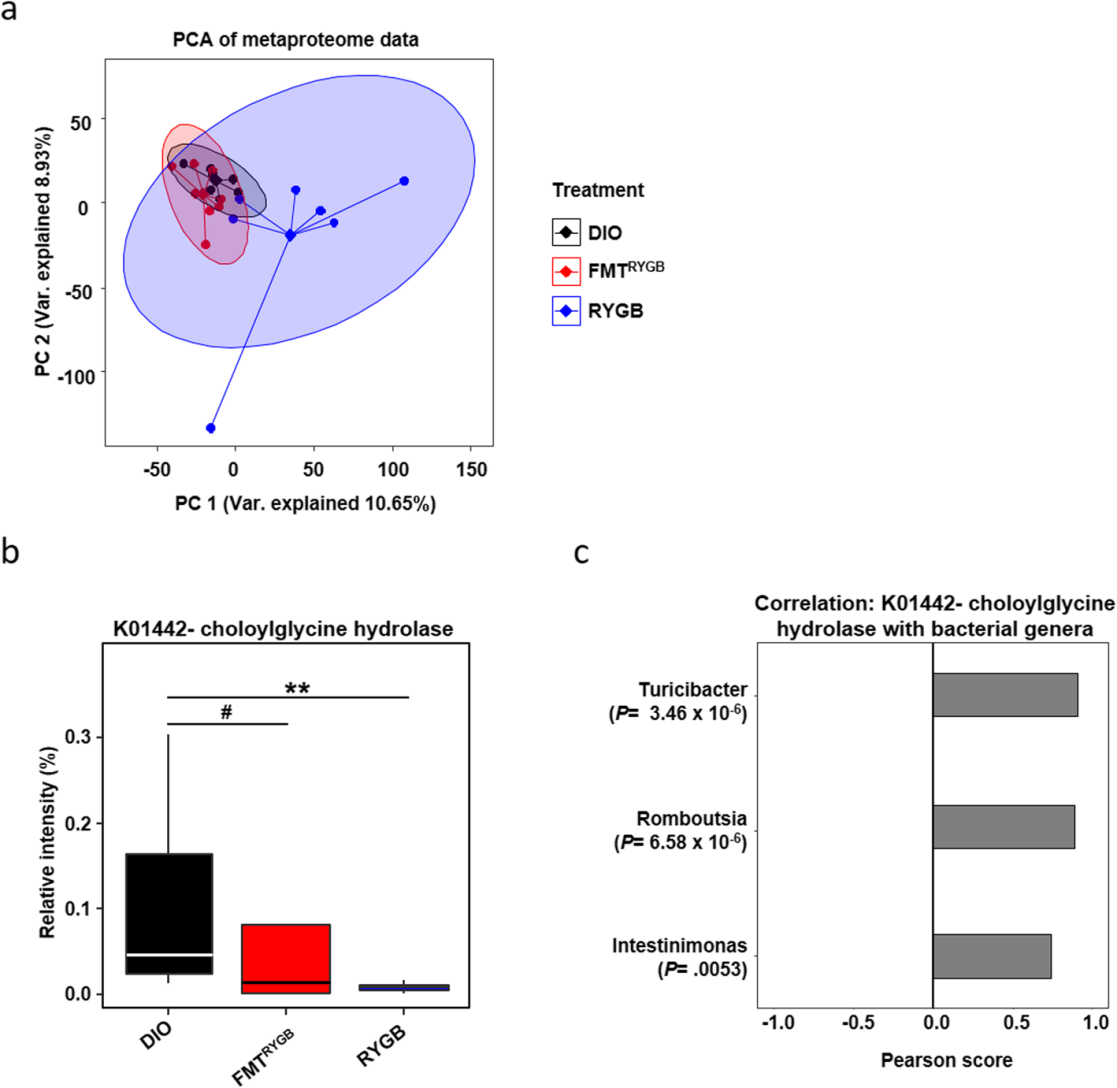
Metaproteomics of intestinal microbiota. **a** Principal component analysis (PCA) of protein group abundances reveals significant differences (PERMANOVA *P* = 0.001) in the metaproteomes between treatments. **b** Significant changes (Kruskal-Wallis test *P* = 0.0456) in the abundance of choloylglycine hydrolase function (KEGG K01442), known to deconjugate conjugated bile acids, between treatments in the intestinal microbiome was observed, with the post hoc pairwise analysis revealing a significant change between RYGB and DIO (Dunn test, *P* = 0.0069 (**)), and a trend between DIO and FMT^RYGB^ (Dunn test, *P* = 0.0823 (#)). **c** Microbiome bacteria genera that are significantly associated with the abundance of choloylglycine hydrolase. *n* = 6–10 animals per group with pooled data from 3 independent experiments

To gain deeper insight into the shaped gut microbiota-host interaction at functional level and identify microbial pathways promoting increased T-CBA abundance in FMT^RYGB^, we applied metaproteomics analysis to the caecum content, which enables large-scale characterization of the entire protein complement of the gut microbiota [15]. In essential difference to the high overlap of taxonomic distribution found at genome level (Fig. 4a), principal component analysis of bacterial protein group intensities revealed significant global differences between DIO and RYGB (*p* = 0.002) as well as between DIO and FMT^RYGB^ animals (*p* = 0.001) (Fig. 6a), indicating significant functional changes expressed by the microbiotas.

In total, 12,113 protein groups from the intestinal microbiota were identified in the cecum content. Using Ghostkoala web application from KEGG, we matched the protein groups to KEGG Orthology IDs and mapped these to metabolic pathways. One hundred forty-two pathways were mapped with a minimum coverage of 15% (Supplementary Table S1). Three pathways (‘inorganic ion transport and metabolism’, ‘ribosome biogenesis’, ‘chromosome and associated proteins’) showed significant (adjusted *p* value < 0.05) alterations, while the pathway ‘starch and sucrose metabolism’ just missed the significance level.

Given the significant increase in T-CBA abundance following RYGB microbiota transfer to HF-DIO, we were particularly interested in pathways involved in taurine biosynthesis or degradation or in bile acid conjugation or deconjugation. Interestingly, we found bile salt hydrolase (BSH, KEGG K01442, also known as choloylglycine hydrolase), the key enzyme function involved in deconjugation of taurine-conjugated BAs produced in the liver to free BAs, to be significantly altered. Compared to protein levels in the microbiota of HF-DIO, a lower BSH protein abundance was found in FMT^RYGB^ (*p* = 0.0823) and RYGB animals (*p* = 0.0069) (Fig. 6b). Shifts in the protein abundance of BSH significantly associated with the bacteria genera Turicibacter (*p* < 0.0001), Romboutsia (*p* < 0.0001), and Intestimonas (*p* = 0.0053) (Fig. 6c), indicating their possible involvement in altered BSH protein levels in FMT^RYGB^ recipients.

### RYGB gut microbiota requires intestinal FXR and systemic TGR5 signaling to reactivate BAT thermogenesis and improve glucose control in HFD-induced obesity

Having demonstrated that the increased intestinal FXR activation by RYGB microbiota transfer modulates systemic BA signaling towards improved metabolic health, we sought to test the physiological role of this receptor activation. To this end, we designed an experiment where intestinal FXR signaling was pharmacologically inhibited in FMT^RYGB^ mice by treating animals with the orally available, intestine-specific FXR inhibitor glycine-ß-muricholic acid (Gly-MCA) [43] *versus* vehicle [44].

**Fig. 7:**
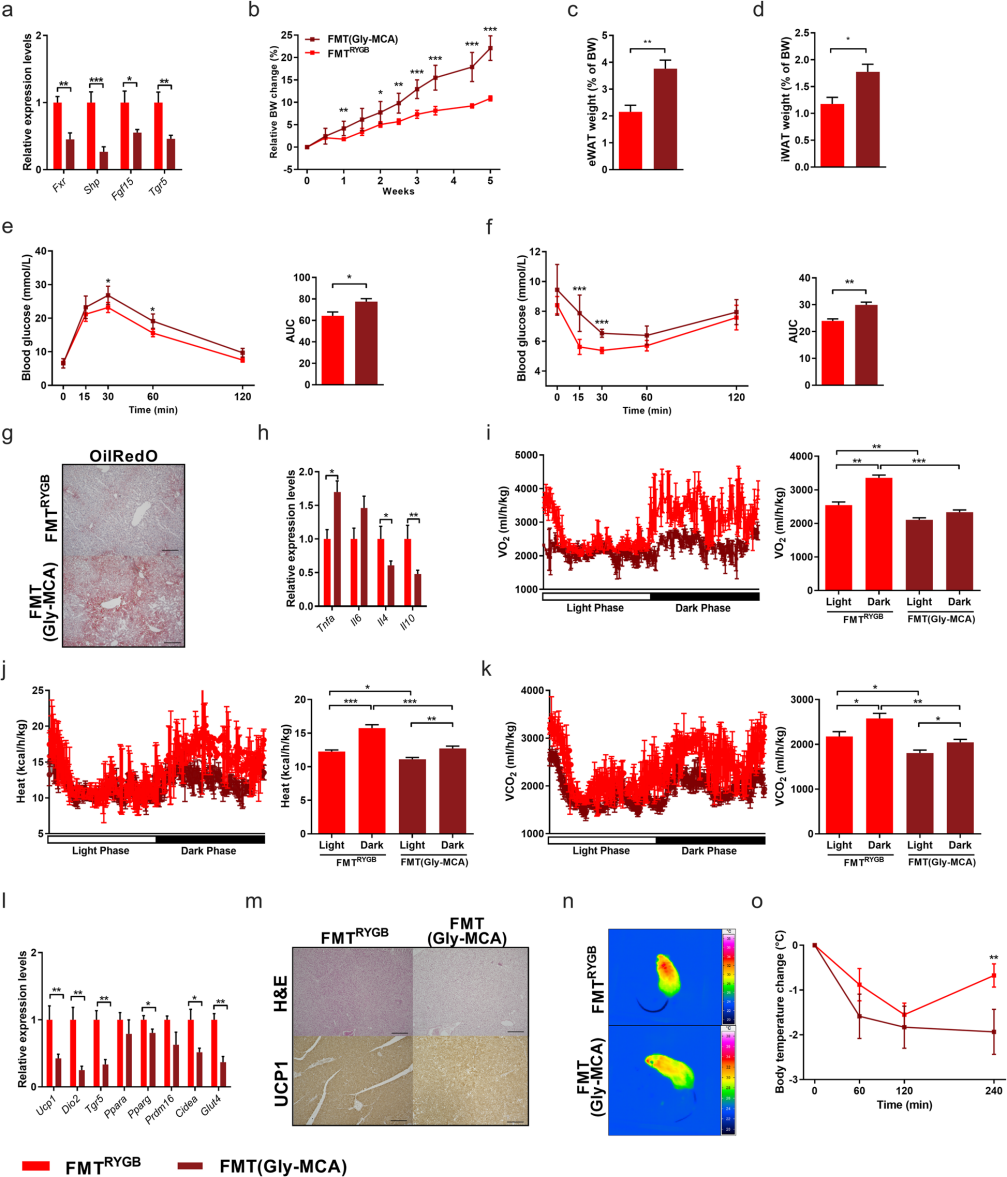
RYGB microbiota requires intestinal FXR and systemic TGR5 signaling to transfer metabolic health benefits in HFD-induced obesity. **a**–**d** Relative mRNA expression of FXR target genes in ileum (**a**), relative body weight change (in %; øBW per group: FMT(Gly-MCA) W0: 29.3 g, W5: 35.7 g; FMT^RYGB^ W0: 28.4 g, W5: 31.4 g) (**b**), and epididymal (eWAT) (**c**) and inguinal (iWAT) white fat mass (WAT) (**d**) relative to body weight of HFD-fed mice at 5 weeks of RYGB fecal microbiota transfer (FMT^RYGB^) co-treated with Gly-MCA (FMT(Gly-MCA)) compared to FMT control mice without co-treatment with Gly-MCA (FMT^RYGB^). **e-h** Oral glucose-tolerance tests (oGTT) (**e**) and insulin-sensitivity tests (ITT) (**f**). OilRedO staining of sections from liver (scale bars 200 μm) (**g**) and hepatic mRNA expression of FXR target genes (**h**). **i**, **k** Oxygen consumption (VO_2_; in ml/(h*kg)) **(i)**, heat (in kcal/(h*kg)) **(j)** and carbon dioxide production (VCO_2_; in ml/(h*kg)) (**k**). **l**–**o** Relative mRNA expression of genes involved in thermogenesis and glucose uptake in BAT (**l**), H&E and UCP1 staining of sections from BAT (scale bars 200 μm) (**m**) as well as infrared images (**n**) and corresponding rectal body temperature (in °C) after 4 h of cold-exposure (**o**). Data are mean ± s.e.m; *n* = 5–10 animals per group with pooled data from 2 to 3 independent experiments. *P < 0.05, **P < 0.01, ***P < 0.001; unpaired two-tailed Student’s *t* test

Suppression of intestinal FXR signaling by Gly-MCA (Fig. 7a) diminished the beneficial effects of RYGB microbiota on improved adiposity (Fig. 7b–d) and glucose homeostasis (Fig. 7e, f) without compromising energy and glucose control in HF-DIO control mice (Supplementary Figure S9). Although Gly-MCA administration did not affect FXR signaling in the liver (Supplementary Figure S10a), it clearly diminished the beneficial effects of RYGB microbiota transfer on hepatic steatosis (Fig. 7g–h and Supplementary Figure S10b), without inherently affecting liver morphology (Supplementary Figure S9e, f). These data indicate that RYGB microbiota-mediated liver protection does at least partly require intestinal, but not hepatic FXR activation.

As the adiposity-reducing properties of RYGB microbiota transfer appear to be clearly dependent on intact gut FXR signaling, but only partly attributable to suppressed appetite (Supplementary Figures S5 and 10c), we further investigated the role of intestinal FXR signaling in accelerated metabolic rate and increased adipose tissue thermogenesis following RYGB microbiota transfer to HF-DIO recipients.

Notably, Gly-MCA treatment clearly reduced the accelerated metabolic rate in FMT^RYGB^ mice (Fig. 7i–k), which was associated with lower thermogenic gene expression (Fig. 7l), reduced amounts of mitochondrial UCP-1 protein in iBAT (Fig. 7m) and WAT depots (Supplementary Figure S10d) as well as lower body temperature and cold tolerance (Fig. 7n, o).

Furthermore, and consistent with the postulated entero-protective effects of intestinal FXR activation, Gly-MCA prevented the beneficial effects of RYGB microbiota on mucosal defense (Supplementary Figure S8c-f) and epithelial barrier integrity (Supplementary Figure S8g–i).

We finally sought to investigate the pathway through which activated FXR signaling in the intestine upon RYGB microbiota transfer regulates improved body energy and metabolic control. Our present findings of pronounced increase in plasma secondary T-CBA (Fig. 5c–e) together with enhanced systemic *Tgr5* expression in FMT^RYGB^ (Fig. 5o) points towards a stimulated systemic TGR5 activation as a possible molecular target of the reprogrammed metabolism following RYGB microbiota transfer. To address this hypothesis, in a separate experimental design, we treated *Tgr*5^−/−^ mice on HFD with rat RYGB microbiota over 35 days. Interestingly, in this global *Tgr5*-depleted model, RYGB microbiota transfer largely failed to facilitate adiposity and appetite control (Supplementary Figure S11a, b), to improve glucose tolerance or systemic insulin sensitivity (Supplementary Figure S11c, d) and lost its stimulating effect on adaptive thermogenesis as demonstrated by unchanged cold tolerance (Supplementary Figure S11e, f), and metabolic rate compared to *Tgr*5^−/−^ mice on HFD (Supplementary Figure S11g–i).

Together, these findings provide strong evidence that intestinal *Fxr* and systemic *Tgr5* are critical molecular targets for RYGB microbiota transfer to attenuate adiposity and metabolic derangements in the obese host.

## Discussion

Despite extensive studies in recent years about the role of the gut microbiota in the development of obesity and related metabolic disorders, little is known about its implications on adiposity reduction and recovery of metabolic health. In particular, the discussed role of the gut microbiome in the outcome of bariatric surgical intervention and the mechanistic gateway to improved adiposity and metabolic control remain poorly understood.

This study highlights two interdependent, yet inherently distinct implications for host-microbiome changes after RYGB in adiposity reduction and multiorgan crosstalk. Firstly, it demonstrates that procedure-specific changes in the metabolism and community composition of the gut microbiota secondary to RYGB intervention are functionally *necessary* to take full effect of the surgery on adiposity reduction, feeding suppression and metabolic improvement. These systemic benefits of RYGB are lost upon microbial depletion by antibiotics, thereby challenging the widespread belief of mechanical restriction and/or malabsorption as central determinants of therapeutic success. Secondly, we here provide first evidence that the repeated transfer of RYGB gut microbiota exerts potent reduction of adiposity and systemic metabolic improvements in a conventional model of HF-DIO, and that these effects are essentially coupled to enhanced activation of thermogenic adipose tissue and suppressed appetite. Together these data suggest that metabolic shifts of the RYGB gut microbiome may be considered not only *necessary*, but also *sufficient* to convey major beneficial metabolic effects of the surgical intervention by fecal transfer.

The loss of increased fat appetite in our RYGB FMT model is notable, as failure of appetite control is a major aspect of weight (re)gain and obesity relapse [19]. As these beneficial effects in our study were specific to fecal transfer from RYGB and permanent even in conventionally raised obese recipients without antibiotic pretreatment, these results provide first evidence for dominant anti-obesogenic properties of the RYGB microbiota over dysbiosis, which may open the path towards novel microbiome-based therapies in human obesity.

Though several neural and hormonal adjustments are certainly involved in the retuned inter-organ cross-talk that governs improved energy and metabolic balance after RYGB [19], we here identify a critical role for modulated BA signaling, probably resulting from a change in metabolic activity in a cluster of bacteria, which is transferable to phenocopy fundamental aspects of surgery success from the operated into the un-operated organism.

More specifically, we demonstrate that RYGB microbiota transfer into HF-DIO animals promotes increased hepatic taurine and BA synthesis *via* the alternative pathway, enhanced enterohepatic BA reabsorption, and reduced microbial protein expression of the BA de-conjugating enzyme BSH, which together encourage enhanced intestinal and systemic T-CBA pool size and BA-receptor signaling.

With this regard, our data on metaproteomics and microbial composition suggest the involvement of selected bacterial genera in regulation of BSH abundance and BA conjugation status, a finding that is of particular interest as the bacterial species *Turicibacter* and *Romboutsia ilealis* have recently been linked to increased susceptibility to obesity in mice [45]. Moreover, by using quantitative trait locus (QTL) analysis, Kemis and colleagues could recently show that the abundance of *Turicibacter* species and levels of plasma cholic acid are both associated with the genomic locus harboring the gene that encodes the ileal bile acid transporter, *Slc10a* [46]. These data further emphasize the intimate link between host genetics, the gut microbiome and host homeostasis, where BA signaling appears to be an important mediator of mutual communication.

Similar to cold exposure [38], microbiota-directed shifts in the BA synthetic pathway and profile, as we here demonstrate towards a dominant alternative pathway and an increase in T-conjugates of chenodeoxycholic acid (CA) derivatives, may essentially regulate the relative activation of intestinal FXR and systemic TGR5 receptor signaling. Indeed, we show that changes in BA composition and pool size following RYGB microbiota transfer activate intestinal FXR-FGF19 signaling in the obese recipient, an effect that is reversible by antibiotic treatment and specific to the transfer of RYGB other than lean microbiota (data not shown).

Notably, the attenuated weight gain and metabolic benefits attributed to RYGB microbiota transfer were clearly compromised in Gly-MCA-treated and in *Tgr5*^*−/−*^ mice, suggesting that intestinal FXR and subsequent systemic TGR5 activation may play an important role in mediating selective aspects of RYGB microbiota transfer on improved energy homeostasis and metabolic control in HF-DIO recipients.

As a ligand-activated nuclear receptor that regulates hepatic BA biosynthesis, transport, and secretion, FXR is involved in multiple metabolic diseases [33, 47]. In agreement with our results, specific intestinal FXR activation by 6-ethyl-chenodeoxycholic acid in rats and by fexaramine in mice [40] has previously been shown to protect against the development of obesity and glucose intolerance, which was associated with enhanced thermogenesis and increased browning of WAT depots [40]. Further in line with our data, FXR was previously reported as a molecular target for weight loss and improved glucose control after bariatric surgery in mice [48], although the gut microbiome as a possible metabolic mediator had been neglected in that study.

Additionally, RYGB-operated patients reveal increased postprandial BA and FGF19 response compared with obese controls [11, 49, 50] which, as we show here, are transmissible signaling responses through fecal transfer.

Interestingly, systemic treatment with fexaramine, while robustly inducing systemic FGF15 release in mice, failed to display major beneficial parts of gut-specific FXR activation [40]. This is of special note, as these findings argue that the FXR-mediated effects on enhanced energy expenditure and glucose control largely depend on the shaped gut bacterial metabolic activity and downstream modulated BA metabolism and receptor signaling. In this light, orally administered fexaramine was reported to substantially increase the relative amount of systemic lithocholic acid (LCA) [40], a potent endogenous ligand for the BA receptor TGR5, which is known to directly modulate adipose tissue thermogenesis by promoting intracellular thyroid hormone activation [37], and promote GLP-1 release from intestinal L cells [51].

More recent findings from [52] complement this emerging picture by identifying hypothalamic TGR5 signaling as an additional key regulator counteracting diet-induced obesity in mice by top-down neuronal mechanisms that convey feeding suppression and activation of adrenergic dependent BAT thermogenesis.

Taken together, these findings may indicate a coordinated systemic signaling crosstalk between both BA receptor systems [42] cooperating to control energy homeostasis and metabolic responses at multiple levels.

Consistent with these reported data, our study demonstrates that RYGB microbiota transfer is also characterized by a strong increase in taurine-conjugated secondary BA species, which predominantly act as endogenous agonists for TGR5 [53]. These pronounced changes in BA composition coincided with enhanced postprandial GLP-1 release, increased expression of *Tgr5*, deiodinase type 2 (*Dio2*)-downstream signaling in adipose tissue, and substantially reduced effects of RYGB microbiota transfer on feeding suppression, energy expenditure, and metabolic control in *Tgr5*^*−/−*^ mice.

Furthermore, and in line with the intimately linked systemic concept, tempered effects of microbiota transfer on adiposity, appetite suppression and metabolic improvement coincided in Gly-MCA-treated mice with a clear downregulation of intestinal and systemic TGR5-DIO2 signaling.

Together, our findings strongly indicate that enhanced signaling crosstalk between intestinal FXR and systemic TGR5 may play an essential role in the adiposity-lowering and metabolic health-mediating effects of RYGB microbiota transfer in the obese recipient, where adipose tissue thermogenesis may relay satiation signal to the brain [54].

Of note, previous studies on mice with intestine-specific *Fxr* deletion or pharmacological inhibition reported protection against obesity and hepatic steatosis when fed a HFD [43, 55]. These apparently contradicting findings underlines the complexity of the dual role of intestinal FXR as a regulator as well as sensor of microbial signaling and host metabolism through different pathways, where similar metabolic traits may result both from reducing and from increasing intestinal FXR signaling in a context-specific manner [33].

While our study reveals a critical role for the RYGB gut microbiota in protecting against adiposity and metabolic distress in rodent models of obesity, it is noteworthy that BA composition varies between rodents and humans with respect to conjugated species [56]. To translate our findings and the emerging knowledge on BA physiology derived from model organisms into clinical benefits, it will be essential for future studies to clarify how specific adaption of bacterial metabolic networks to distinct bariatric surgical procedures may affect downstream BA receptor signaling pathways and subsequent host homeostatic responses in human obesity.

Encouragingly, our present findings are supported by first clinical pilot data, revealing that enhanced abundance of plasma and fecal secondary BAs is associated with increased intestinal transit and improved peripheral insulin sensitivity in patients beneficially responding to RYGB fecal transfer [57]. Future FMT studies will have to elaborate on these clinical pilot data in a probably more ingenious study design to provide novel translational and essential mechanistic insights.

## Conclusions

Combining metabolic profiling and omics technologies with a carefully designed mechanistic assessment in rodent models of obesity, our findings provide important novel insights as to the mechanistic basis of the gut microbiota in energetic and metabolic health benefits retrieved by RYGB surgery.

Here, we demonstrate that shifts in microbial composition and metabolic activity are indispensable for RYGB intervention to completely unfold its effects on adiposity reduction and systemic metabolic reprogramming, and that these effects can be prevented by gut microbiota depletion. Moreover, expanding the focus from causality to therapy, we show that critical features of the RYGB-like phenotype can be transferred by fecal transplantation and identify microbiome-modulated taurine metabolism and downstream systemic BA signaling as molecular targets reactivating adipose tissue thermogenesis and improving glucose control in obese recipients via the systemic circulation. This work highlights the critical importance of the gut microbiota and the multiorgan crosstalk in modulating energy and metabolic control, and strengthens the rationale for microbiota-targeted strategies in the quest for effective long-term weight management solutions.

## Methods

### Animals and diets

Animal experiments followed the international guideline of animal care and were approved by the local governmental authority for animal care (state directorate of Saxony, Germany). All experiments were performed at least three times in independent approaches. Age-matched (8–10 weeks old) male Wistar rats (managed under the International Genetic Standardization/IGS program) and C57BL/6J WT mice (purchased from Charles River, Sulzfeld, Germany) were allocated to experimental groups based on their body weight, which was measured at the same circadian time throughout the experiments. TGR5 knockout (KO) mice were kept on a C57BL/6 background and were kindly provided by Verena Keitel (Heinrich-Heine-University, Düsseldorf, Germany) [58]. Heterozygous animals were used for breeding to obtain littermate TGR5 knockout and wild-type animals. Animals were individually housed on a 12-h light/dark cycle in facilities with an ambient temperature of 21–23 °C and 40–60% humidity. If not stated otherwise, animals were fed standard chow (SC) (EF V1534-000, Ssniff Spezialdiäten GmbH) or high-fat diet (HFD) containing 58% of total energy as fat, 25.5% as carbohydrate, and 16.5% as protein (EF D12331, Ssniff Spezialdiäten GmbH). Allocation into experimental groups and schematic overview of the study design is presented in Supplemental Material and Supplemental Figure S1. For feeding studies with the intestine-specific FXR antagonist Gly-MCA (Biomol), mice were administered with the compound added to bacon-flavored HFD (D12331, Ssniff Spezialdiäten GmbH) at a concentration of 250 mg/kg body weight. Control mice received the same bacon-flavored HFD without the compound. *TGR5*^*−/−.*^ and WT littermates were fed a HFD (Research diet D12331, Ssniff Spezialdiäten GmbH), starting at 8 weeks of age. Animals developing serious health issues (e.g., atypical, dramatic weight loss, behavioral abnormalities), as individually judged by trained animal keepers and veterinarians, were excluded from analysis.

### In vivo metabolic experiments

For the oral glucose tolerance test, animals were fasted overnight followed by an oral gavage of 20% dextrose (1 g/kg BW). Blood glucose was determined at 0, 15, 30, 60, and 120 min after glucose challenge (AccuChek Guide, Roche). Insulin tolerance tests were performed after overnight fast with Insulin administered intraperitoneally (0.5 U/kg BW). Core body temperature was measured with a clinical rectal thermometer (Thermalert model TH-5; Physitemp). Body temperatures were read after 6-h cold exposure (8 °C) with a VarioCAM Jenoptik infrared camera (InfraTec GmbH). Calorie uptake was calculated via oxygen bomb calorimetry. Estimated total energy expenditure (TEE) was calculated by energy balance method (see Supplementary Information).

For metabolic phenotyping (PhenoMaster, TSE-Systems), inter-species microbial transplantation from rat donors into mouse recipients was performed according to the same protocol as for intra-species transfer. Efficacy of the inter-species transfer was monitored carefully and gave comparable results as for rats. All parameters were measured continuously and simultaneously. The integrity of the gut barrier was tested after a 4-h day-fast by oral gavage of 0.6 g/kg BW of fluorescein isothiocyanate (FITC)-dextran (FD4; Sigma-Aldrich) and measurement of the fluorescence in plasma samples collected 60 min later. If not otherwise stated, animals were fasted for 4 h before harvesting.

### Seahorse analysis of murine brown adipocytes

Stromal vascular fraction was isolated from newborn (unknown sex) mice as described previously [22]. Briefly, excised interscapular BAT was digested with collagenase for 30 min at 37 °C and filtered twice using nylon meshes of 100 μm and 30 μm, respectively. Brown pre-adipocyte fraction was re-suspended and seeded on a 6-well plates. The pre-adipocytes were then immortalized with SV40 large T antigen and unselected cells were cultured, expanded up to 4 passages and cryopreserved. For Seahorse analysis, adipocytes were seeded in appropriate 24-well plates (Agilent) at 40,000 cells/well in 100 μl of adipocyte growth medium (DMEM supplemented with 10% FBS and 0.1% penicillin/streptomycin). The following day, differentiation was induced with induction medium (growth medium, 20 nM insulin, 1 nM triiodothyronine, 1 μM dexamethasone, 100 μM 3-isobutyl-1-methylxanthine, 1 μM rosiglitazone, and 125 nM indomethacin). After 2 days, the medium was replaced with brown adipocyte differentiation medium (growth medium, 20 nM insulin and 1 nM triiodothyronine). The following day, cells were treated with 50% (vol/vol) of rat serum (or PBS as control) for 6 h and the Seahorse XF Cell Mito Stress Test (Agilent) was performed according to manufacturer instructions. The following substances were used: norepinephrine (1 μM, Sigma-Aldrich), oligomycin (2 μM, Sigma-Aldrich), carbonyl cyanide-4-(trifluoromethoxy)phenylhydrazone (FCCP, 1 μM, Tocris), rotenone (0.5 μM, Tocris), antimycin A (0.5 μM, Tocris), Hoechst staining (10 μg/ml, Sigma-Aldrich). As all pre-treatments were assessed in one run, control treatment (PBS instead of serum) was used as reference for all serum treatments, i.e., DIO, FMT^RYGB^, RYGB, RYGB(ABx). The number of cells labelled with Hoechst staining was calculated with Cytation 5 Cell Imaging Multi-Mode Reader for normalization.

### Statistics

All statistical analyses were performed by the GraphPad Prism software. Data are expressed as mean ± s.e.m unless otherwise stated. Comparisons between two groups were performed using a non-paired two-tailed Student’s *t* test with no assumption of equal variance. ANOVA with post hoc Tukey correction was used for comparison between multiple groups as indicated. Numbers per group in the figure legends refer to the number of animals per group. Individual data points were only excluded if technical issues were detected during the analysis procedure. Statistical significance is indicated by *p* value (*P < 0.05, **P < 0.01, ***P < 0.001). The statistics regarding microbiome analysis is described in the subsection on microbiome analysis (see above).

Specific information about plasma analyses, real-time PCR, histology, immunofluorescence, Western blotting, electron microscopy, microbiome analysis, metaproteomics, and mass-spectrometric measurements are given in the Supplemental Material 1.

## Supplementary Information

**Additional file 1: Supplementary Figure S1.** Schematic of experimental design.

**Additional file 2: Supplementary Figure S2. **Lean microbiota depletion has no effect on host energy and glucose control.

**Additional file 3: Supplementary Figure S3.** Antibiotics treatment affects the host’s fecal microbiota abundance, food preference and mitochondrial respiration.

**Additional file 4: Supplementary Figure S4.** Lean microbiota transfer has no effect on host energy and glucose control in HFD-induced obesity.

**Additional file 5: Supplementary Figure S5.** Reduced fat appetite is not the main factor for lower adiposity and improved host metabolism secondary to RYGB microbiota transfer into HFD-induced obesity.

**Additional file 6: Supplementary Figure S6.** Post-RYGB gut microbiota reduces adipose tissue fibrosis.

**Additional file 7: Supplementary Figure S7.** Post-RYGB gut microbiota alters intestinal metabolites in HFD-induced obesity.

**Additional file 8: Supplementary Figure S8.** Post-RYGB gut microbiota improves small intestinal health by reducing inflammation, permeability and apoptosis.

**Additional file 9: Supplementary Figure S9. **Gly-MCA per se shows no effect on energy and glucose control in HFD-induced obesity.

**Additional file 10: Supplementary Figure S10.** Gly-MCA does not affect hepatic FXR signaling but activates WAT browning.

**Additional file 11: Supplementary Figure S11.** 5 RYGB microbiota transfer (FMT) largely fails to counter adiposity and affect metabolism in TGR5^-/-^ knockout mice.

**Additional file 12: Supplementary Table S1.** Pathway analysis.

## Data Availability

The mass spectrometry metaproteomics data have been deposited to the ProteomeXchange Consortium via the PRIDE partner repository [59] with the dataset identifier PXD026606. 16S RNA gene sequencing data was deposited in the SRA repository [60] and can be found under BioProject: PRJNA735921. Metabolomics mass spectrometry data are available at the NIH Common Fund's National Metabolomics Data Repository (NMDR) website, the Metabolomics Workbench, https://www.metabolomicsworkbench.org where it has been assigned Project ID (PR001157).
