# Supplementary figures and images for: Functional changes of the gastric bypass microbiota reactivate thermogenic adipose tissue and systemic glucose control via intestinal FXR-TGR5 crosstalk in diet-induced obesity
Source: Microbiome. 2022 Jun 24;10:96. doi: 10.1186/s40168-022-01264-5 (PMC9229785; doi:10.1186/s40168-022-01264-5)

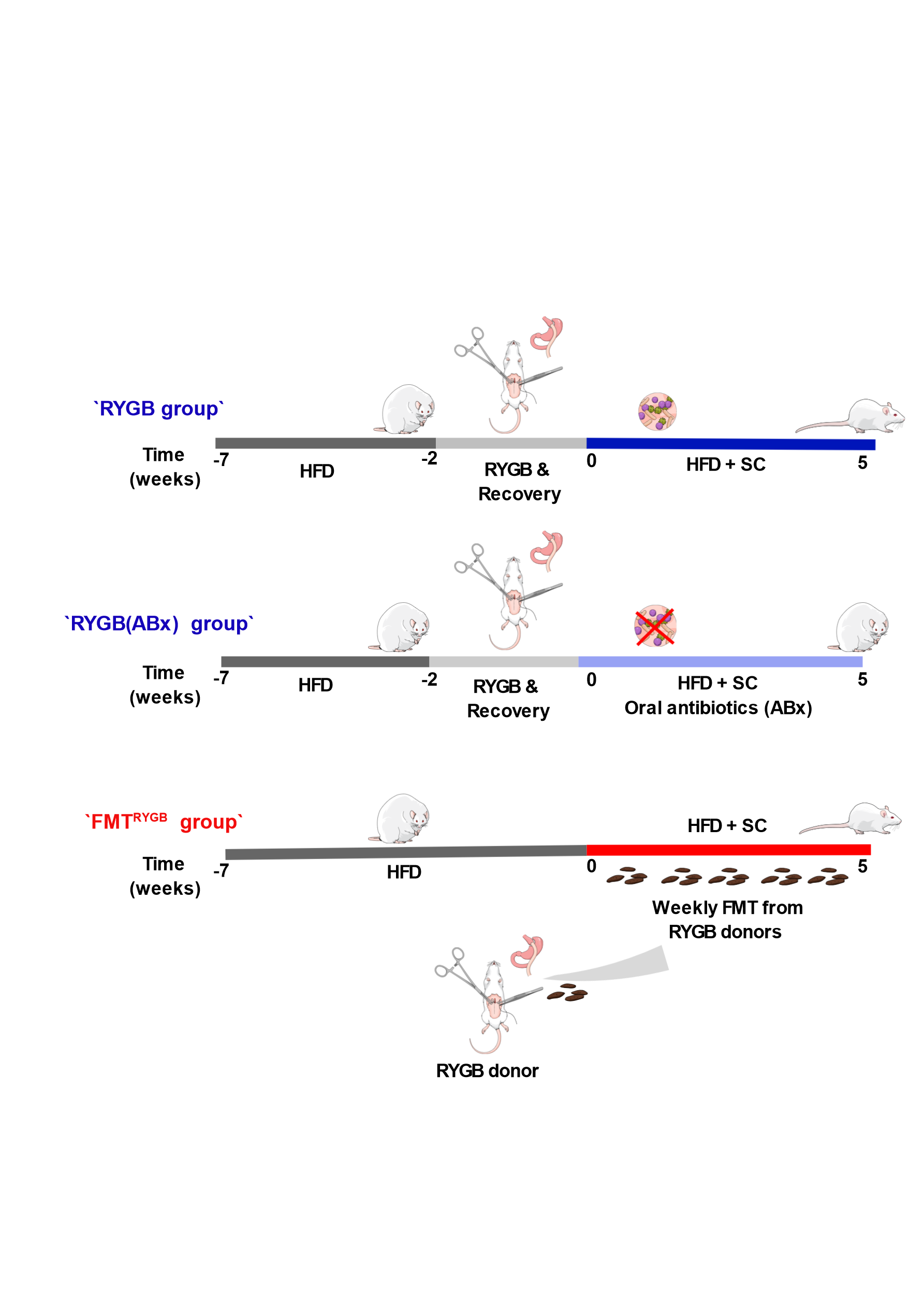

Supplement: Supplementary file 2 — Additional file 1: Supplementary Figure S1. Schematic of experimental design. [file 40168_2022_1264_MOESM1_ESM.tiff]

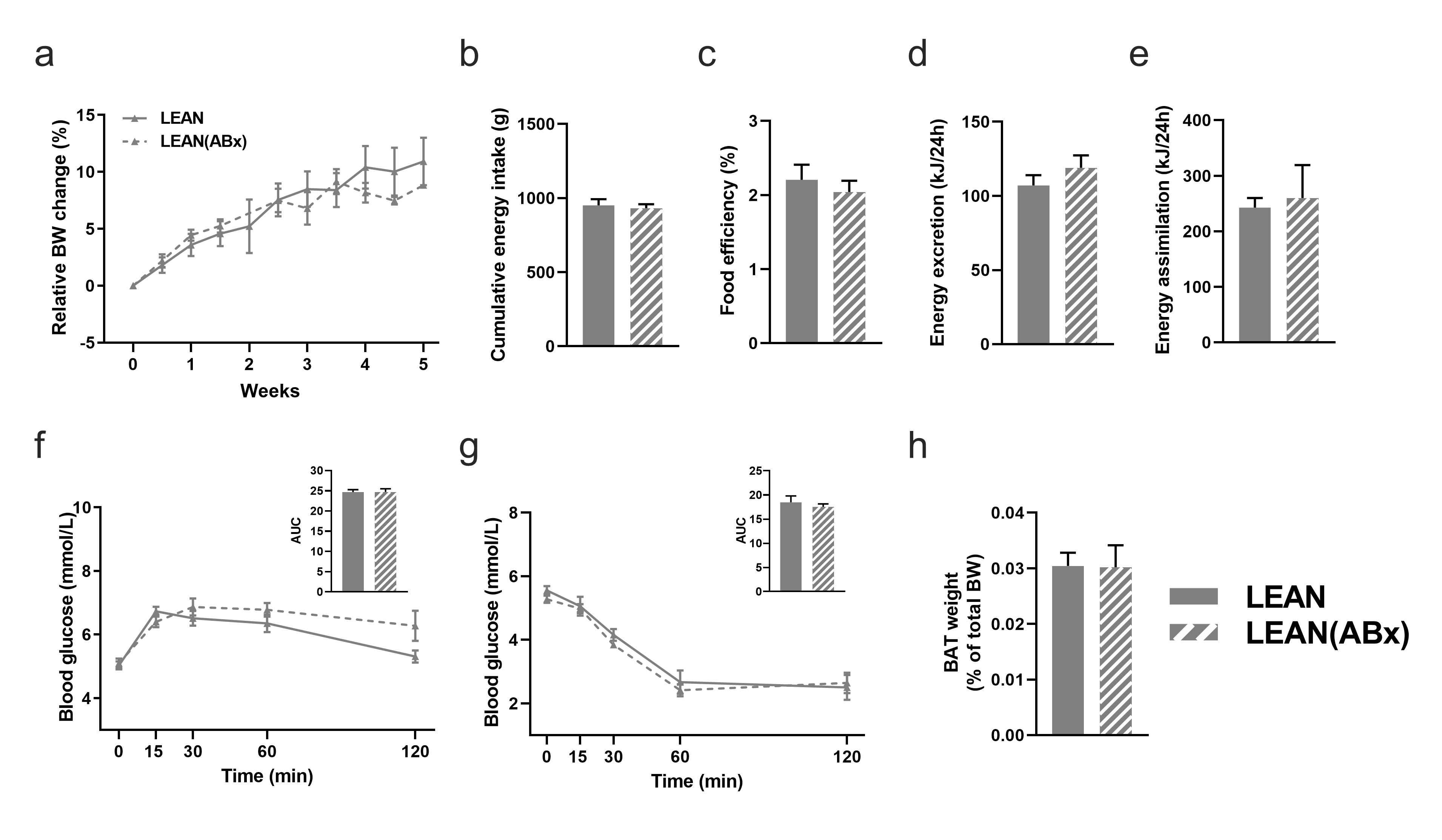

Supplement: Supplementary file 3 — Additional file 2: Supplementary Figure S2. Lean microbiota depletion has no effect on host energy and glucose control. [file 40168_2022_1264_MOESM2_ESM.tif]

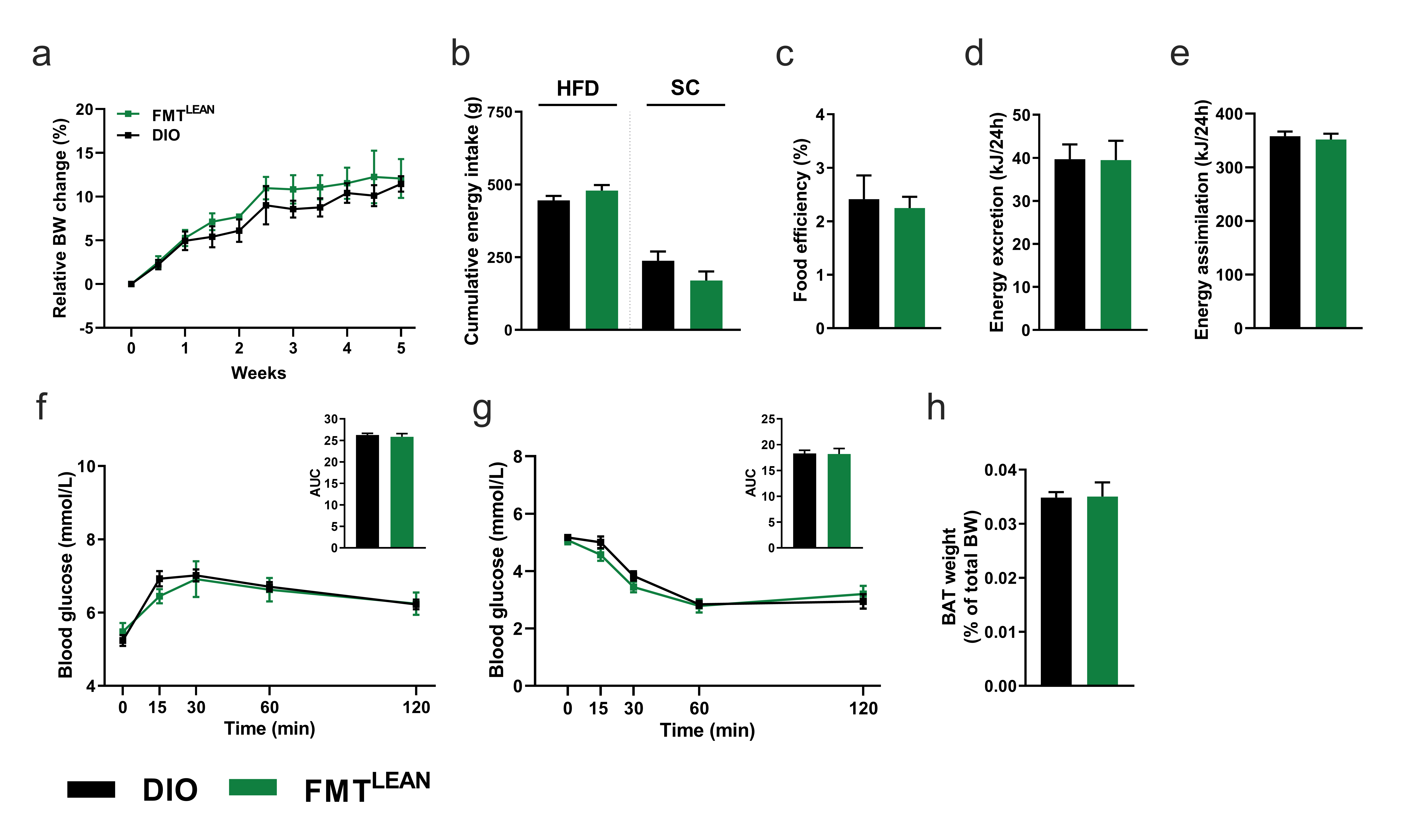

Supplement: Supplementary file 5 — Additional file 4: Supplementary Figure S4. Lean microbiota transfer has no effect on host energy and glucose control in HFD-induced obesity. [file 40168_2022_1264_MOESM4_ESM.tif]

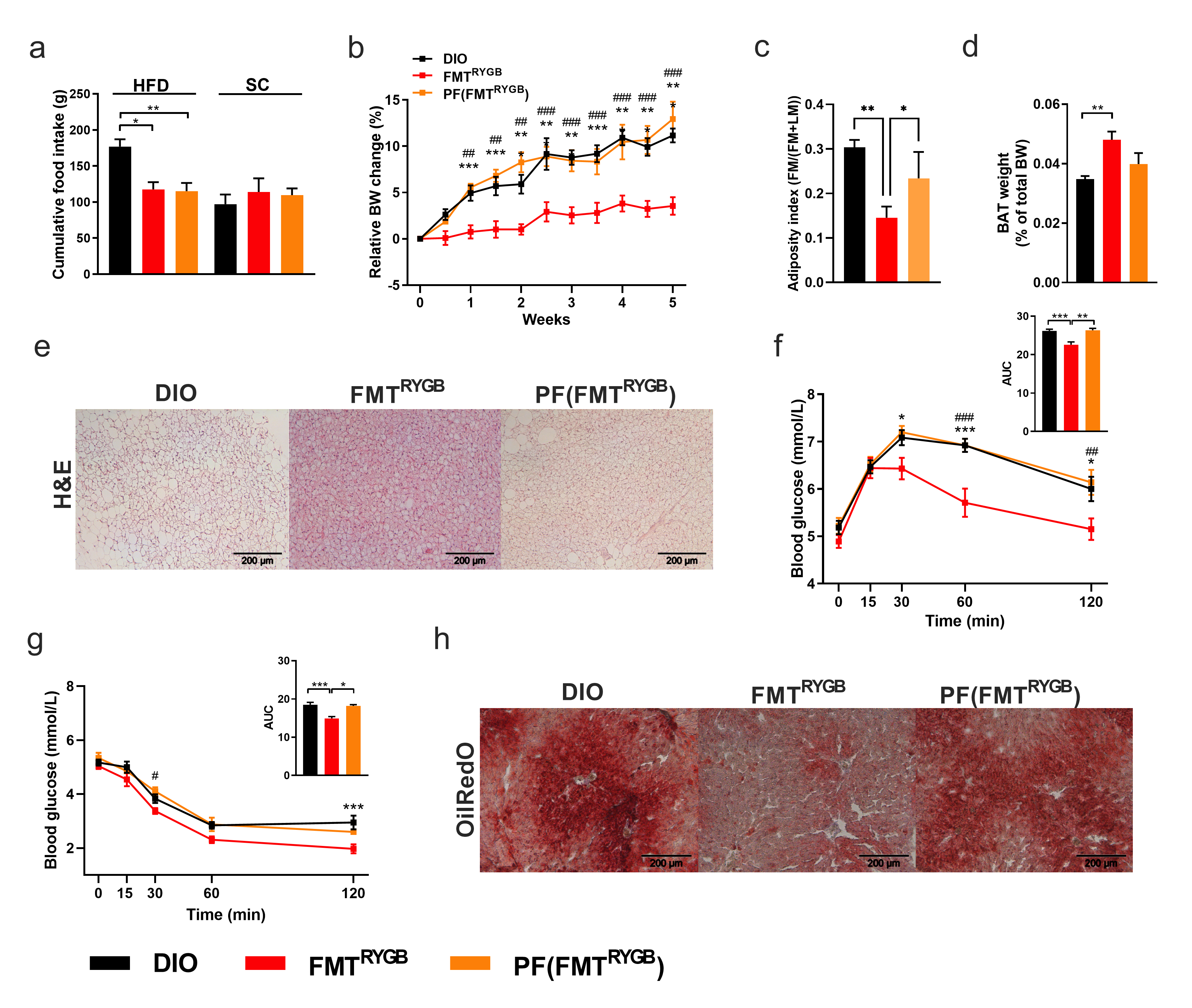

Supplement: Supplementary file 6 — Additional file 5: Supplementary Figure S5. Reduced fat appetite is not the main factor for lower adiposity and improved host metabolism secondary to RYGB microbiota transfer into HFD-induced obesity. [file 40168_2022_1264_MOESM5_ESM.tif]

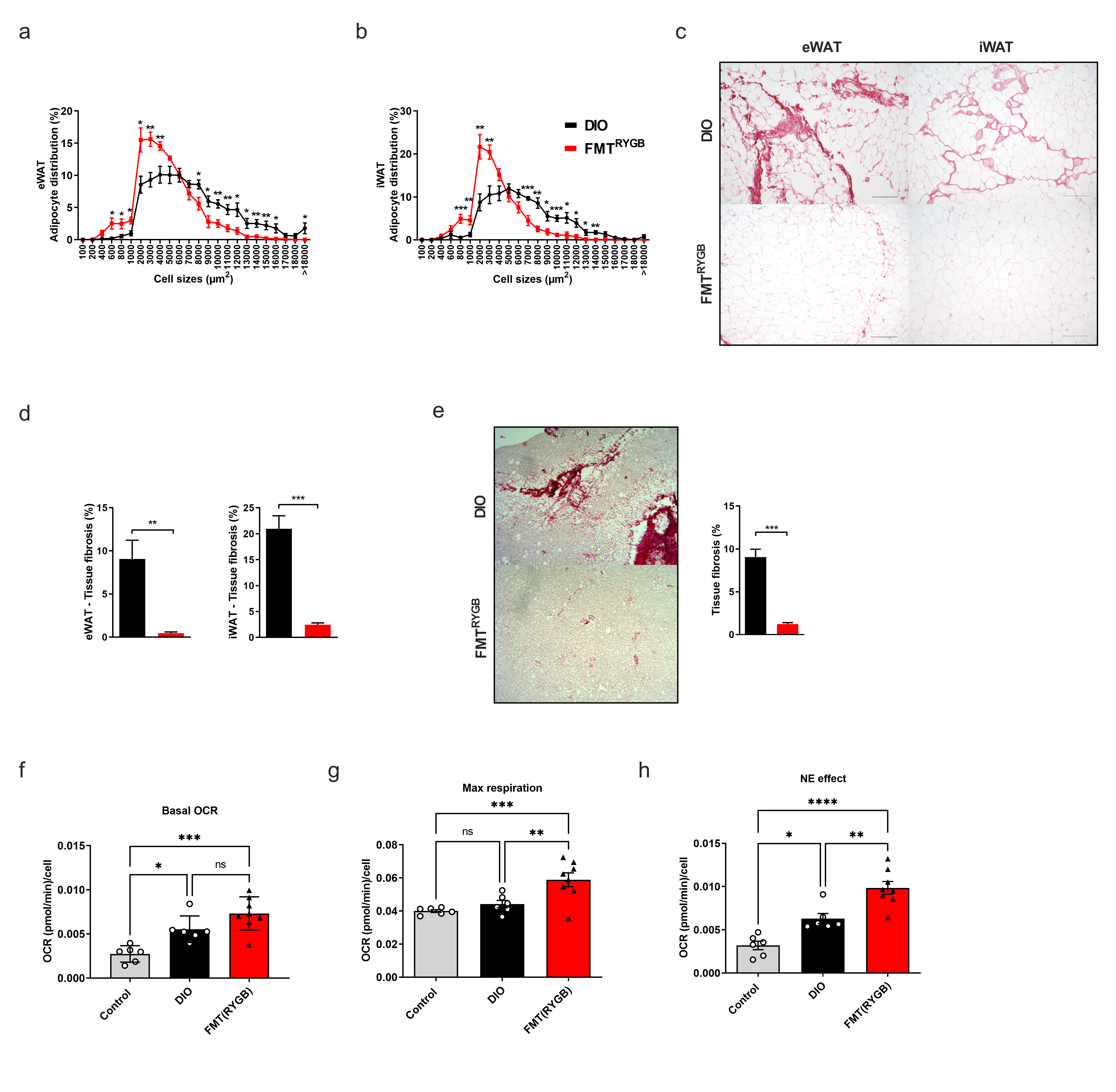

Supplement: Supplementary file 7 — Additional file 6: Supplementary Figure S6. Post-RYGB gut microbiota reduces adipose tissue fibrosis. [file 40168_2022_1264_MOESM6_ESM.tif]

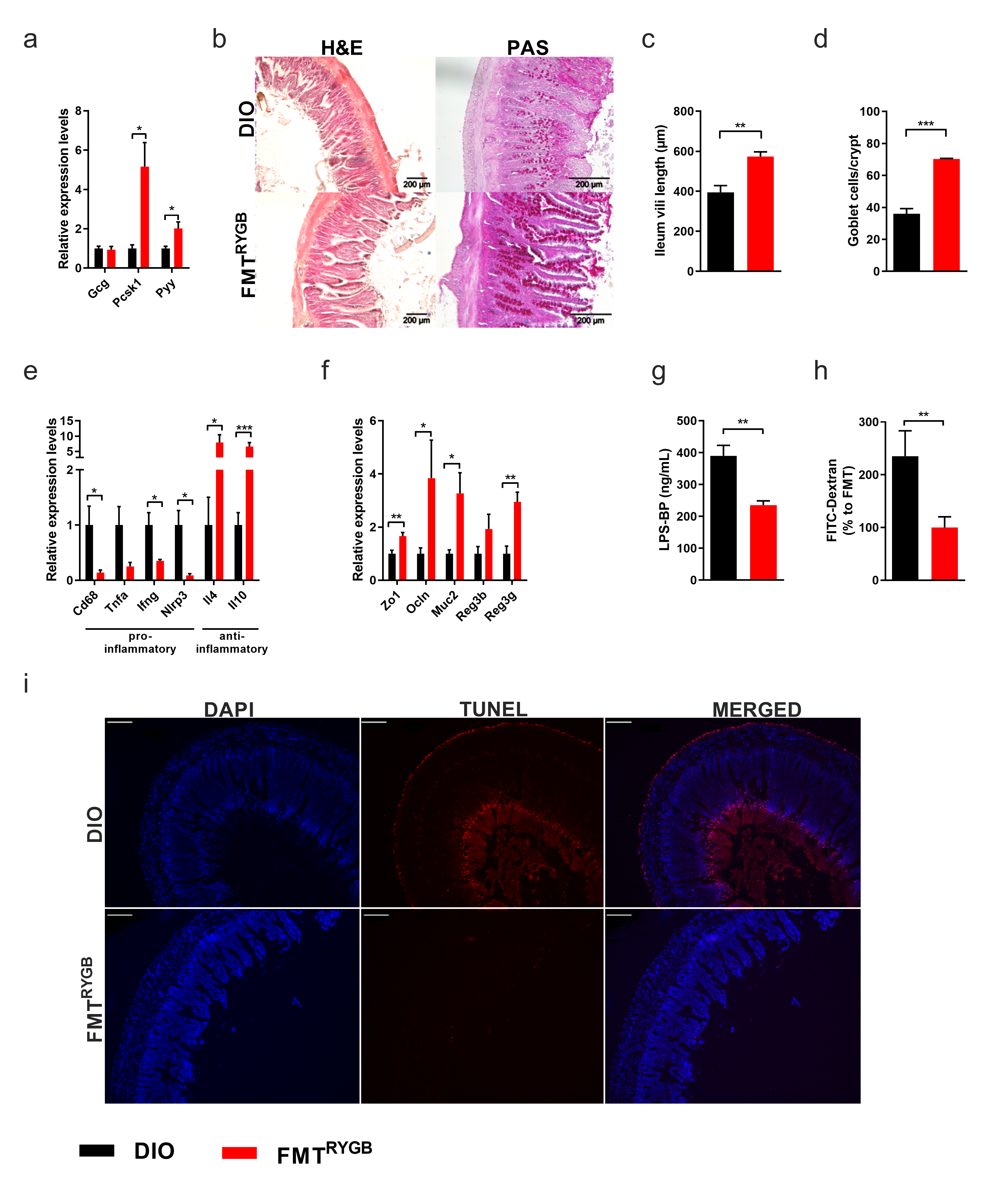

Supplement: Supplementary file 9 — Additional file 8: Supplementary Figure S8. Post-RYGB gut microbiota improves small intestinal health by reducing inflammation, permeability and apoptosis. [file 40168_2022_1264_MOESM8_ESM.tif]

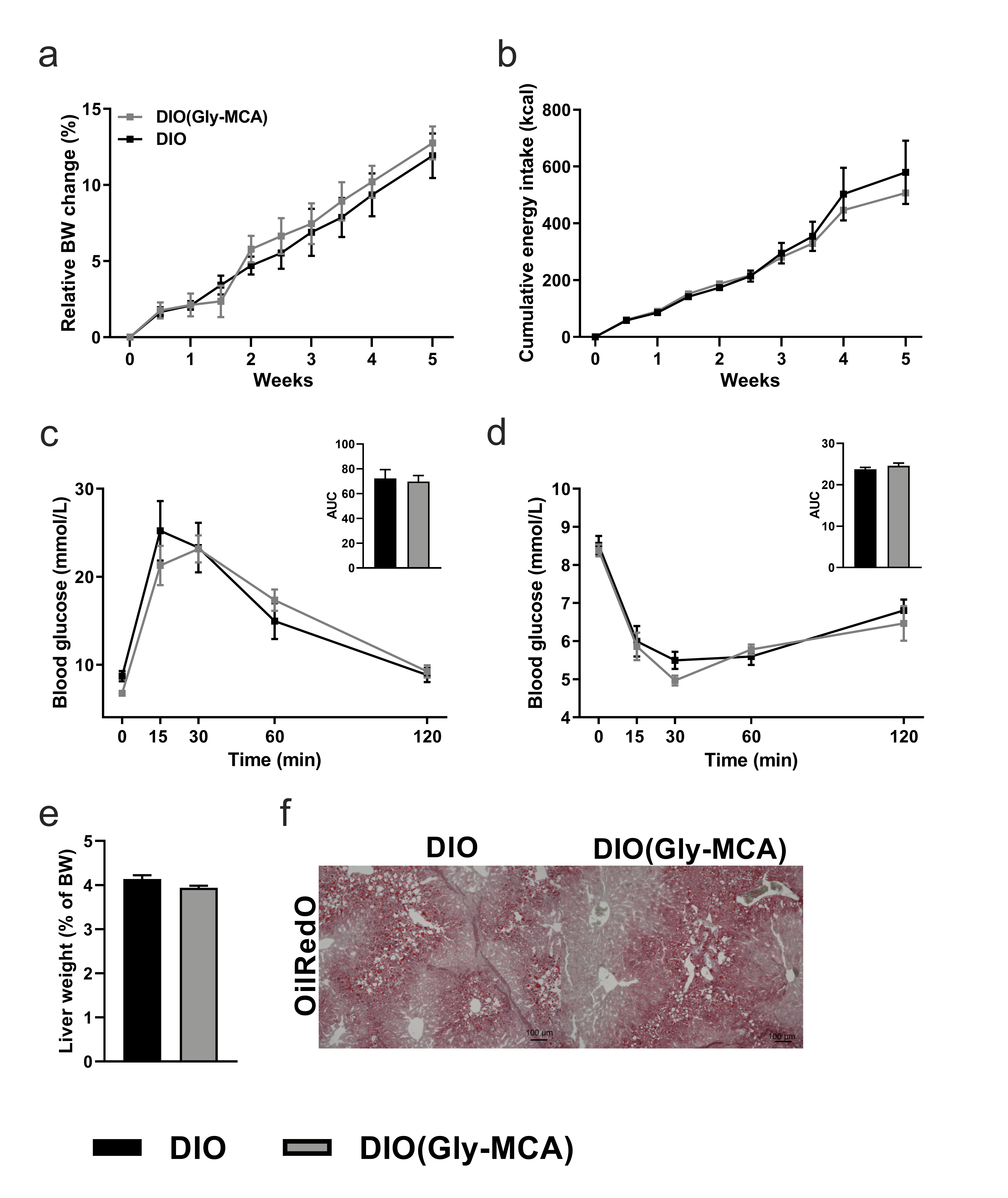

Supplement: Supplementary file 10 — Additional file 9: Supplementary Figure S9. Gly-MCA per se shows no effect on energy and glucose control in HFD-induced obesity. [file 40168_2022_1264_MOESM9_ESM.tif]

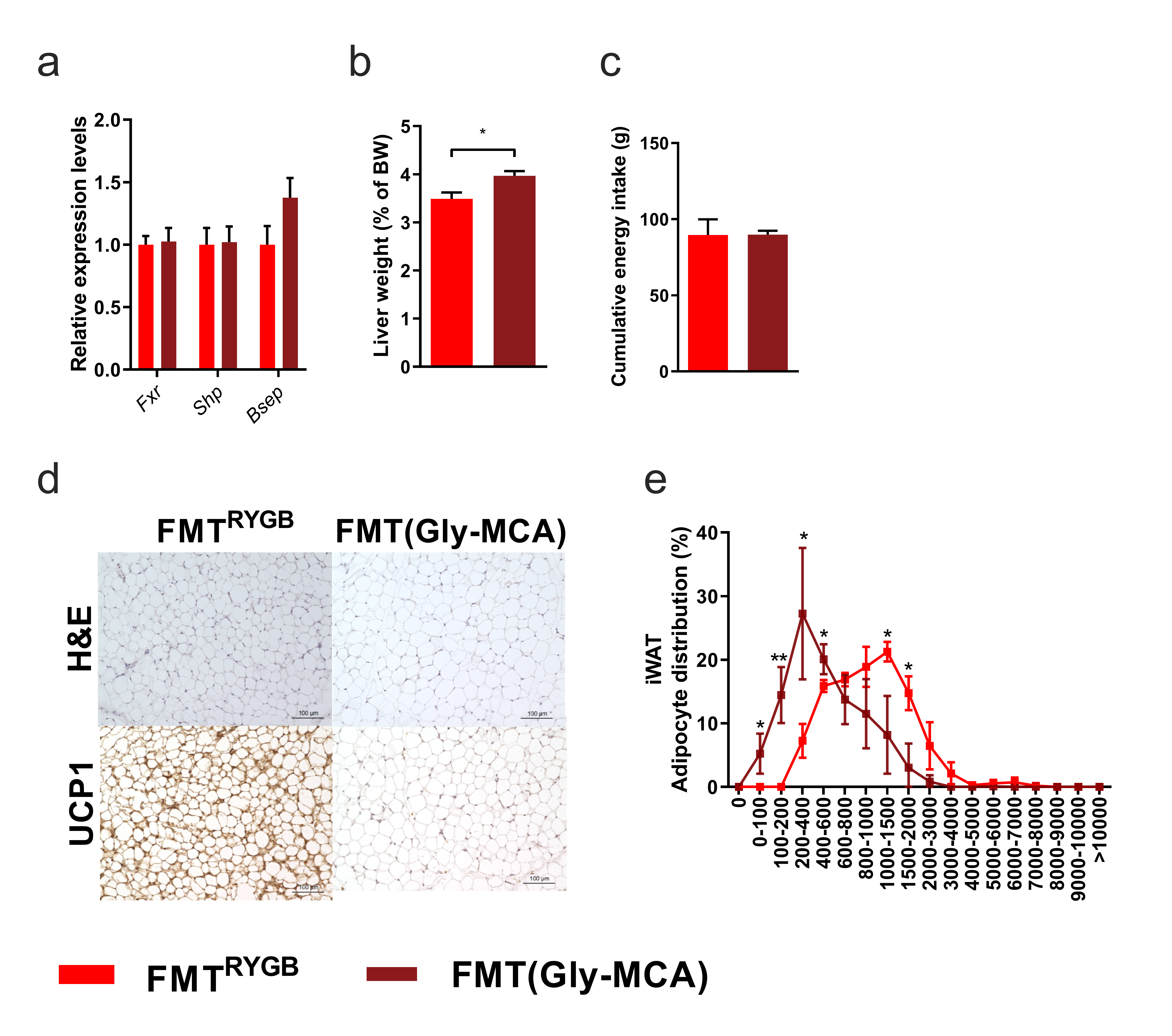

Supplement: Supplementary file 11 — Additional file 10: Supplementary Figure S10. Gly-MCA does not affect hepatic FXR signaling but activates WAT browning. [file 40168_2022_1264_MOESM10_ESM.tif]
