# Supplementary material for: Functional changes of the gastric bypass microbiota reactivate thermogenic adipose tissue and systemic glucose control via intestinal FXR-TGR5 crosstalk in diet-induced obesity
Source: Microbiome. 2022 Jun 24;10:96. doi: 10.1186/s40168-022-01264-5 (PMC9229785; doi:10.1186/s40168-022-01264-5)
Supplement: Supplementary file 13 — Additional file 12: Supplementary Table S1. Pathway analysis. [file 40168_2022_1264_MOESM12_ESM.docx]

**Functional changes of the gastric bypass microbiota reactivate thermogenic adipose tissue and systemic glucose control via intestinal FXR-TGR5 crosstalk in diet-induced obesity.**

**Supplemental Material I**

**Material and Methods**

**Experimental groups for rat experiments.**

1. Rats in the ‘RYGB group’ underwent RYGB surgery according to a previously published protocol (1). Postoperatively, animals received a two-choice diet, consisting of a SC and HFD. Once the animals recovered and achieved a stabilized weight reduction at 2 weeks postoperatively, body weight and food intake were recorded daily.
2. Rats allocated into the ‘RYGB(ABx) group’ were handled the same as animals in the ‘RYGB group’. From postoperative week 2 onwards, animals received an antibiotic treatment (ABx) consisting of ampicillin (1 g/l; Ratiopharm, Germany), vancomycin (0.5 g/l; Ratiopharm), neomycin (1 g/l; Bela-pharm, Germany), and metronidazole (1 g/l; CP-Pharma, Germany), provided freshly every day via drinking water (2, 3). All antibiotics were given for a time period of 5 weeks. (4).
3. Rats in the ‘LEAN group’ served as healthy, age-matched controls, constantly kept under SC *ad libitum*.
4. Rats in the ‘LEAN-ABx group’ received the same antibiotic treatment as animals in the RYGB-ABx group, serving as a control for antibiotic-specific side effects. All antibiotics were given for a time period of 5 weeks.
5. Rats in the ‘DIO group’ received sham surgery but were otherwise handled like RYGB littermates.
6. Rats in the ‘FMT^RYGB^ group’ received fecal RYGB microbiota transplantation once per week, but were otherwise handled like DIO littermates. For fecal transplantation experiments, 100 mg of fresh stool from bodyweight-stabilized RYGB donors (from week 2 postoperatively) was re-suspended in 1 ml of PBS, homogenized carefully and administered via oral gavage with 200 μl of the suspension.
7. Rats in the ‘FMT^LEAN^ group’ were handled like DIO littermates, but were orally gavaged with 200 μl of filtrate received from lean donors, and served as a control for fecal transplantation and specificity of effect size(s) as a function of the donor microbiota.
8. Rats in the ‘PF(FMT^RYGB^) group’ were handled like DIO littermates, but pair-fed to the SC/HFD food intake of FMT^RYGB^ littermates, serving as a control for effects secondary to reduced caloric intake.
9. TGR5^-/-^ knock out mice in the `TGR5^-/-^(DIO) group received sham gavage with PBS analogous to their FMT littermates.
10. TGR5^-/-^ knock out mice in the `TGR5^-/-^(FMT) group` received fecal RYGB microbiota transplantation from RYGB donor rats twice per week , but were otherwise handled like TGR5^-/-^(DIO) littermates. The oral gavage was similar to description from ´FMT^RYGB^ group´ and served as a control for fecal transplantation and specificity of effect size(s) as a function of the donor microbiota in a TGR5^-/-^ knock out mice model.

**Total energy expenditure (TEE).** Energy expenditure was calculated as described by *Chevalier et al.* (5). The following formula was used:

*TEE (kcal/g) = energy intake (kcal/g) – 9.4 x Δfat mass + 1.8 x (kcal/g) x Δfat-free mass*

Differences in fat and lean mass were calculated from two DXA scan measurements during the first and last week of treatment. 9.4 and 1.8 kcal/g are empirical values for energy content of fat and lean mass, respectively.

**Determination of energy assimilation.** To measure feed intake and to collect feces for bomb calorimetry, animals were set on grid ﬂoors for 48 h after 4 weeks of intervention.

The energy content in feces and diets was determined by bomb calorimetry (IKA C5003; IKA Werke, Staufen, Germany) according to *Weitkunat et al.* (6). Feces were carefully cleaned from bedding or diet, and lyophilized (Alpha 1-4 apparatus; Christ Gefriertrocknungsanlagen GmbH, Germany) overnight. Subsequently, 1 g of each sample was ground in a mortar, pressed into tablet form, weighed into a glass crucible and then completely combusted in the bomb calorimeter. Experimental diets were used without prior drying and grounding. Assimilated energy was calculated by subtracting energy loss via feces (kJ/day) from diet energy intake (kJ/day).

**Plasma analyses.** Plasma lipids (triglycerides, total cholesterol, free glycerol) were determined using colorimetric assay (Sigma-Aldrich), plasma cytokine levels were quantified using V-PLEX Proinflammatory Panel 2 Rat multiplex assay (Meso Scale Diagnostics), and ELISA was used to quantify LPS-BP (Hycultec), insulin (DRG), GLP-1 (Merck) and PYY (Crystal Chem), respectively, according to the manufacturer’s instructions.

**Real-time PCR.** Total RNA was extracted from frozen tissues using TRIzol (Invitrogen) and standard chloroform extraction protocol. Reverse transcription of mRNA into cDNA was performed using QuantiTect Reverse Transcription Kit (Qiagen). Expression levels of target genes were quantified by quantitative RT-PCR using Taqman expression assays (Thermo Fisher Scientific) or SYBR green (Roche). Quantification of gene expression was always normalized to a reference gene and a list of primer sequences is given in the table below:

| **Primers for rat tissues** | | |
| --- | --- | --- |
| **Gene** | **Forward primer** | **Reverse Primer** |
| **Actb** | GGAGATTACTGCCCTGGCTCCTA | GACTCATCGTACTCCTGCTTGCTG |
| **Adrb3** | ACTCACCGCTCAACAGGTTT | TTCTGGAGAGTTGCGGTTCC |
| **Agrp** | TGCTAGATCCACAGAACCGC | GCAAGGTACCTGTTGTCCCA |
| **Asbt** | AAGGCTGAGTTTCTGAGTTCTT | CTCCTGCATTATTTTCTTGTGGGAA |
| **Atgl** | GCTCTGTTTTCCCCAGGCTT | AACTCCACACCCTACTCAC |
| **B2m** | ACATCCTGGCTCACACTGAA | ATGTCTCGGTCCCAGGTG |
| **Baat** | CGTAGTTCGACAGCAACCCT | TGCTTGGTTGAGGAGGGAAA |
| **Bsep** | TCCAACCCCTGTAGAACCCT | CAGCGGAATGGAAGCATGTG |
| **Cartpt** | TGAGAAGGAGCTGCCAAGG | CGGAATGCGTTTACTCTTGAGC |
| **Cd68** | CTTCCCACAAGCAGCACAG | AATGATGAGAGGCAGCAAGAGA |
| **Cdo** | TTCCTTAGCGGCGGATTTTG | TCCTTTTCCTCAGCATCACCC |
| **Cidea** | TTCCTCGGCTGTCTCAATGT | GCCCGCATAAACCAGGAAC |
| **Cidec** | AGAAAGCCCAGCTATCCCTT | CCAGGAACTGCTGCATGTAA |
| **Cyp7a1** | GCTTTACAGAGTGCTGGCCAA | CTGTCTAGTACCGGCAGGTCATT |
| **Cyp7b1** | AGCTCTGGTGTTCTCAGTGC | GAGAGGCTTTGACAGGTGCT |
| **Cyp8b1** | GCAAGGAGAGGCAGAGGCAA | GCAAGGAGAGGCAGAGGCAA |
| **Cyp27a1** | ACTTGCAGAGGGTAGTCCCA | TGGGGACACTTACAGGGGAT |
| **Dio2** | GCACAGGAGACTGACTGAGG | AATTTAACCTGTTTGTAGGCGTC |
| **Fgf19** | CGGACCCTGTCGTGTTAGT | TCGGCAACCTCCAAAGTCA |
| **Fxr** | CAGCAGACCCTCCTGGATTA | TCTTCGTGGTCCAGTGTCTG |
| **Glut4** | CAACTGGACCTGTAACTTCATCG | ACGGCAAATAGAAGGAAGACGTA |
| **Hprt** | ACAGGCCAGACTTTGTTGGA | TGCCGCTGTCTTTTAGGCTT |
| **Hsl** | CCCCGAGATGTCACAGTCAAT | GAATTCCCGGATCGCAGAA |
| **Lpl** | GAGATTTCTCTGTATGGCACA | CTGCAGATGAGAAACTTTCTC |
| **Mch** | GACCACAAAGAACACAGGCTC | CTGGTCCTTTCAGAGCGAGG |
| **Muc2** | AGCCGAGGTTGTTACTGTGG | GGTGTTGTGGTATGGGGTGT |
| **Npy** | TAACAAACGAATGGGGCTGT | TGTCTCAGGGCTGGATCTCT |
| **Ocln** | GTGACCAGTGACATCAGCCA | TGGGTTTGAATTCATCCGGC |
| **Pgc1a** | CACCAAACCCACAGAGAACAG | GGTGACTCTGGGGTCAGAG |
| **Pomc** | GAAGGTGTACCCCAATGTCG | CTTCTCGGAGGTCATGAAGC |
| **Ppara** | TAGCAACAATCCGCCTTTTGT | GCGATCAGCATCCCGTCTT |
| **Pparg** | ACAAGGACTACCCTTTACTGAAATTACC | GTCTTCATAGTGTGGAGCAGAAATGCTG |
| **Reg3b** | CCCTCTGCACGCATTAGTTG | AGGCCAGTTCTGCATCAAACC |
| **Reg3g** | GTGTGGATTGGGCTCCATGA | CAGAAATCCTGAGGCTCGTGT |
| **Shp** | TGATGGCTCCCAAAACCTCC | CTGAAGATTGGTTGGCCCCT |
| **Slc51a** | GAAGGCAAAGGCATACAGAGC | GCCTTACATCTTGCTGCTTCC |
| **Slc51b** | GAGACCCCAGATGCCCAC | ACCAAGGGTGCTTACTACTGTTT |
| **TauT** | GTGGTGAGTATGTGGAGGTTG | GCGGGCATCACAAGACCT |
| **Tgr5** | GTGCTTCGAGGAAGACCCAA | AGTCCAAGTCAGTGCTGCAT |
| **Zo1** | AAGGGGAAACCCGAAACTGA | CGGAATTGCCTTCACTCTGG |
|  |  |  |

| **Primers for mitochondrially encoded genes (rat tissues)** | | |
| --- | --- | --- |
| **Gene** | **Forward primer** | **Reverse Primer** |
| **Actb** | GGAGATTACTGCCCTGGCTCCTA | GACTCATCGTACTCCTGCTTGCTG |
| **mt-Co1** | CACAGTAGGGGGCCTAACAG | GCTATGATGGCGAATACTGC |
| **mt-Cytb** | CGGCTGACTAATCCGATACC | TGGGAGTACATAGCCCATGA |
| **mt-Atp6** | CCACACACCAAAAGGACGAA | CATAGGGGGATGGCTATGCT |
| **mt-Atp8** | TGCCACAACTAGACACATCCA | TGTGGGGGTAATGAAAGAGG |
| **mt-Nd2** | GTCACCCAAGGAATTCCCCTA | GAAATTGCGAGAATGGTGGT |
| **mt-Nd3** | GGCTTCGACCCAACAAGTTC | GGCAGTTGCTATTATTGTAGTGG |

| **Taqman assays for rat tissues** | | |
| --- | --- | --- |
| **Gene** |  |  |
| **Foxp3** | Rn1525092 |  |
| **Fgf21** | Rn00590706 |  |
| **Ifng** | Rn00594078 |  |
| **Il4** | Rn01456866 |  |
| **Il10** | Rn99999012 |  |
| **Nlrp3** | Rn04244620 |  |
| **Pcsk1** | Rn00567266 |  |
| **Gcg** | Rn00562293 |  |
| **Pyy** | Rn01460420 |  |
| **Tnfa** | Rn99999017 |  |
| **Ucp1** | Rn00562126 |  |
|  |  |  |
| **Primers for mouse tissues** | | |
| **Gene** | Forward Primer | Reverse Primer |
| **Bsep** | CAATCATCCGTAACTTTGTTGCC | GCATTCTTGGTGTGGAAGTCAAAA |
| **Cd68** | CCCACCTCCCATTTCTCCTT | AGTCAGGTTTGGGGTGGGAT |
| **Cidea** | GCCGTGTTAAGGAATCTGCTG | TGCTCTTCTGTATCGCCCAGT |
| **Dio2** | CAGTGTGGTGCACGTCTCCAATC | TGAACCAAAGTTGACCACCAG |
| **Fgf15** | GCCATCAAGGACGTCAGCA | CTTCCTCCGAGTAGCGAATCAG |
| **Fxr** | TGGGCTCCGAATCCTCTTAGA | TGGTCCTCAAATAAGATCCTTGG |
| **Glut4** | GTGGCTCTGCTGCTGCTGGAACG | GCGGGGGCCCTGGCTGAAGAG |
| **Mcp1** | TTAAAAACCTGGATCGGAACCAA | GCATTAGCTTCAGATTTACGGGT |
| **Ppara** | CGGGAAAGACCAGCAACAAC | TGACTGGCGCTGACATATCG |
| **Pparg** | CGTGAAGCCCATCGAGGACATC | TGGAGCAGGGGGTGAAG |
| **Prdm16** | AATGCTGTGGATGCCTGACA | CTCGTCCCCCTTCTGTCCTA |
| **Shp** | TCTGCAGGTCGTCCGACTATTC | AGGCAGTGGCTGTGAGATGC |
| **Tgr5** | CTGTGTGAGATCCGCCGAC | CGACGCTCATAGGCCAAGA |
| **Ucp1** | CCGAAACTGTACAGCGGTCT | CCGAGAGAGGCAGGTGTTTC |
|  |  |  |
| **Taqman assays for mouse tissues** | | |
| **Gene** |  |  |
| **Il4** | Mm00445259 |  |
| **Il6** | Mm00446190 |  |
| **Il10** | Mm01288386 |  |
| **Tnfa** | Mm00443258 |  |

**Histology, immunofluorescence and Western blotting (WB).** Tissues were extracted, fixed in 4% paraformaldehyde (Sigma), paraffin embedded (except for Oil Red O staining, which was performed on frozen sections) and cut in 6 μm thick sections and stained with H&E using standard techniques. For histologic examination of pancreatic islets, the whole paraffin-embedded pancreas was cut consecutively and stained alternatingly by Hematoxylin/Eosin (H&E) stain and by immunofluorescence staining for insulin. Islets were sized and counted on alternated sections spaced of 60 µM using ImageJ. Periodic acid-Schiff staining (PAS) was accomplished following manufacturer’s (Sigma-Aldrich) instructions. Cell counts and measurements of sizes and lengths were carried out using ImageJ. Immunohistochemistry was done using rabbit anti-UCP1 (Sigma-Aldrich, #ab23841) and rabbit anti-FGF19 (antikoerper-online, #ABIN6257192). Immunofluorescence staining was performed for insulin (C27C9) (Cell Signaling, #3014) and FXR(NR1H4) (Biozol, #PRS-27-918) following the manufacturer’s recommendations. Apoptosis was determined using Click-iT TUNEL assay (Thermo Fisher Scientific). All images were taken with a Keyence Fluorescence Microscope BZ-X800. For WB, snap-frozen BAT samples were grinded and prepared for SDS–PAGE using a RIPA buffer supplemented with protease and phosphatase inhibitors (Roche). 15 μg of total lysate was loaded on each lane and subsequently transferred on a nitrocellulose membrane. Blocking was performed with 5% BSA for 1 hour at room temperature. Phosphorylated and total HSL were detected with specific antibodies (pHSL(Ser660): Cell Signaling, #cs4126, dilution 1:1000; total-HSL: Abcam, #ab45422, dilution 1:500), incubated over night at 4°C. Horseradish peroxidase (HRP)-conjugated antibodies were used as secondary antibodies. Protein bands were detected with Pierce TM ECL Western Blotting substrate (Thermo Scientific) and chemiluminescence detection method on a G:BOX (Syngene) and quantified with the corresponding GeneTools analysis software (Syngene).

**Electron microscopy.** BAT samples were post-fixed in pH 7.4-buffered 1% osmium tetroxide at room temperature for 1 h, rinsed in cacodylate buffer (pH 7.4), dehydrated in a graded series of acetone including 1% uranyl acetate stain at 70% acetone for 30 min. and subsequently embedded in Durcupan resin (Roth). For structural orientation, semi-thin sections were cut at 1 μm thickness and stained with toluidine blue. Ultrathin sections (50 nm) were cut on an Ultracut II (Leica Microsystems). Sections were examined with a Zeiss LEO 912 Omega (Zeiss) transmission electron microscope at 80 kV and digital micrographs were obtained with a dual speed 2K-on-axis CCD camera based YAG scintillator (TRS-Tröndle).

**Microbiome analysis.**

Frozen caecum content samples were processed for bacterial DNA isolation using QIAamp Stool Mini Kit (Qiagen) according to the manufacturer’s instructions. Total bacterial content was analyzed exemplarily (n=3 animals) by real-time PCR using bacterial 16S rDNA specific primers (bacteria-16S-F: TCCTACGGGAGGCAGCAGT, bacteria-16S-R: GGACTACCAGGGTATCTAATCCTGTT). In short, DNA was isolated from weighted fecal samples and qRT-PCR was performed in triplicate using fixed eluate volumes. Signal intensities were first transformed into expression levels by reversal of the log-transformation and then normalized to used fecal mass by dividing by the correction factor (ratio of fecal masses). Normalized bacterial DNA content (mean of triplicate measurements) was displayed on a logarithmic scale.

For analysis of community composition, bacterial DNA samples were submitted to BGI (Hong Kong) for bacterial 16S DNA V3-V4 amplicon sequencing using the Illumina MiSeq PE300 platform. Following primers were used for library preparation: forward primer: 341F:ACTCCTACGGGAGGCAGCAG; reverse primer: 806R:GGACTACHVGGGTWTCTAAT.

Sequencing resulted in demultiplexed sequencing data. For each sample two files in fastq format were provided, one for the forward reads and one for the reverse reads. Using the DADA2 R-package quality of reads were checked. Forward reads were trimmed at base pair position 280 and reverse reads were trimmed at base pair position 200. DADA2 was used to learn error rates, followed by filtering, denoising, merging of paired reads and constructing of amplicon sequence variants (ASVs). Taxonomy was assigned to ASVs using the DADA2 package in combination with the Ribosomal Database Project (RDP) database (http://rdp.cme.msu.edu/index.jsp).

Normalization of ASV read counts, determination of alpha-diversity indices and calculation of relative abundance for each taxonomic level was accomplished with Rhea. Significant differences in alpha-diversity indices and relative abundance of taxa between sample groups was determined by Kruskal-Wallis Test with Benjamini-Hochberg method for multiple comparisons where appropriate (independent tests >20), followed by posthoc pairwise statistical analysis using the Dunn test. Beta-diversity between samples was analyzed by non-metric multidimensional scaling (NMDS) dissimilarity analysis with global significant differences between sample groups was calculated by PERMANOVA using the Adonis function from the vegan R-package. Figures were constructed using the ggplot2 R-package.

**Mass spectrometric measurements.** Bile acid measurements were carried out with AbsoluteIDQ Bile Acid Kit (Biocrates Life Sciences AG) according to the manufacturer’s instructions. The liquid chromatography-mass spectrometry (LC-MS/MS) analysis was carried out by MRM acquisition on a Waters Acquity UPLC System coupled to a QTRAP 5500 (AB Sciex Concord). Data processing was performed with the provided quantification method Kit (Biocrates Life Sciences AG).

Amino acids, amines and selected lipid groups (lysophosphatidylcholines, phosphatidylcholines and sphingolipids) were analyzed with the AbsoluteIDQ p180 Kit (Biocrates Life Sciences AG). Measurements were performed on a QTRAP mass spectrometer (MS) applying electrospray ionization (ESI) (ABI Sciex API5500Q-TRAP). The MS was coupled to an UPLC (Waters Acquity, Waters Corporation). The metabolites were separated by a hyphenated reverse phase column (Agilent, Zorbax Eclipse XDB C18, 3.0 x 100 mm, 3.5 µm) preceded with a precolumn (Security Guard, Phenomenex, C18, 4 x 3 mm; Phenomenex), applying a gradient identification. Quantification was achieved by multi reaction monitoring (MRM) standardized by applying spiked-in isotopically labelled standards in positive and negative mode. Data processing and metabolite quantification was performed with MetIQ software (Biocrates Life Sciences AG).

For short chain fatty acid (SCFA) quantification, the method described by Han et al.(7) was used with some modifications. Plasma samples were mixed with acetonitrile to a final concentration of 50% acetonitrile and derivatized with 3-nitrophenylhydrazine. The mix was afterwards diluted 1:50 in 10% acetonitrile and injected into the LC-MS/MS system. Chromatographic separation of SCFAs was performed on an Acquity UPLC BEH C18 column (1.7μm) (Waters) using H2O (0.01% FA) and acetonitrile (0.01% FA) as the mobile phases. For identification and quantification, a scheduled MRM method was used, with specific transitions for every SCFA.

For the quantification of cecal content samples, approximately 10 mg of each sample were used. Samples were processed with steel balls and extraction medium (acetonitrile:water; 1:1) in a ball mill to extract metabolites for downstream processing as described for plasma.

**Metaproteomics:**

*Protein extraction and proteolytic cleavage:* Bacterial protein extraction was done by a modified protocol as previously described (8). For each sample, approximately 0.5 g cecal content was resuspended in 1 mL Lysis-buffer (50 mM Tris, 5 mM EDTA, 0.4% SDS, 50 mM NaCl, 1 mM PMSF, pH= 8) and disrupted with a Fastprep (FastPrep -24, MP Biomedicals). Then samples were shaken in a Thermomixer (Thermomixer comfort 5355, Eppendorf) at 1400 rpm, at 60 °C for 15 min. This was followed by sonication using an ultrasound probe (UP50H, Hielscher) and samples were spun at 10,000 rcf at 4°C for 10 min. Supernatants, containing the protein content, were kept and frozen at -20°C for storage.

For the proteolytic cleavage of proteins, protein extract where thawed and 100 µg of protein was precipitated from each bacterial protein extract, separated by SDS-PAGE and further processed by in-gel reduction and alkylation of cysteine residues followed by cleavage of proteins with trypsin, eluting of resulting proteolytic peptides and desalting peptides as previously described (8).

*LC-MS/MS measurements:* LC-MS/MS measurements were done as decribed in Haange *et al.* (9). In short, peptides were reconstituted in 15 µL 0.1% formic acid and 5 µL were injected for separation into nano-HPLC system (UltiMate 3000, Dionex/Thermo Fisher Scientific, Idstein, Germany) coupled online for analysis with an Orbitrap Q Exactive HF mass spectrometer (Thermo Fisher Scientific, San Jose, CA, USA). Peptides were trapped on a C18-reverse phase trapping column (Acclaim PepMap ® 100, 75 µm x 2 cm, particle size 3 µM, nanoViper, Thermo Fisher) and washed for 5 min with 0.1% formic acid with a 5 µL/min solvent flow. Then peptides were eluted using a 90 min linear gradient from 4% B to 25% B (A: 0.1% formic acid in MS-grade water; B: 80% acetonitrile, 0.1% formic acid in MS-grade water) followed by a 30 min gradient from 25% B to 50% B and a 3 min gradient to 99% B with 5 min isocratic flow, then 2 min gradient to 4% B and finally an isocratic flow for 5 min at 4% B. After trapping, during the entire analytical run the solvent flow rate was set to 300 nL/min and the eluting peptides were separated on a downstream analytical column (Acclaim PepMap ® 100, 75 µm x 25 cm, particle size 3 µM, nanoViper, Thermo Fisher) kept at 35°C before being ionized by a Triversa Nanomate (Advion) ion source and entering the Orbitrap Q Exactive mass spectrometer: Q Exactive mass spectrometer settings were as noted in Haange *et al.* (9)

*Bioinformatics, statistics and data analysis for metaproteomics:* Raw spectra data files were processed using Proteome Discoverer version 2.2 (Thermo Fisher Scientific, v2.2, San Jose, CA, USA). Spectra searches were performed by the tandem mass ion search algorithms Sequest HT. The database for spectral searches was constructed by downloading and combining protein sequences from Uniprot repository (www.uniprot.org, September 2020) of all the bacteria genera identified in the 16S rRNA gene sequencing data with protein sequences from the taxa *Rattus norvegicus*. Additional search parameters were tryptic cleavage, maximum of two missed cleavage sites, a precursor mass tolerance threshold of 10 ppm and a fragment mass tolerance threshold of 0.02 Da. Also, carbamidomethylation at cysteines was selected as a static and oxidation of methionine selected as a variable modification. Data were filtered with a peptide FDR set at 1% and only “rank one” peptides were considered for later analysis. Protein grouping was enabled. Label-free quantification of protein group intensities was calculated by averaging the peak areas of the top 3 peptides for protein group using the precursor ions area detector node of Proteome discoverer software.

Protein sequences were loaded into Ghostkoala to determine KEGG orthologous functions (10). Taxa for proteins sequences were retrieved from the header in the corresponding. uniprot fasta entry. In-house written R-scripts were used to annotate a function and lowest common ancestor to each protein group using the information from all the protein sequences assigned to the group. Using a further in-house written R-script, relative abundance of each taxon, KEGG function and KEGG pathway in the microbiome was calculated by summing the normalized intensities of the corresponding protein groups from Proteome Discoverer output and dividing by the summed normalized intensities of all bacterial protein groups and multiplying by 100 to receive a percentage. Analysis of KEGG pathway association was done using in-house written R-scripts which annotate KEGG IDs to pathways using the KEGG-related databases. Statistics were done in R (11). Global differences in the metaproteomes of treatment groups were determined by principal component analysis (PCA) using normalized protein group intensities with significant separation calculated by PERMANOVA using the vegan package in R (12). For multiple group comparisons, the Kruskal-Wallis test with a posthoc Dunn-test was used. Hmisc package in R was used for correlation analysis. Where appropriate, *P* were corrected for multi-testing using the Benjamini-Hochberg method (13). The R package ggplots2 was used to visualize data (14).

**References Supplemental Material:**

1. Hankir MK, Seyfried F, Hintschich CA, Diep TA, Kleberg K, Kranz M, et al. Gastric Bypass Surgery Recruits a Gut PPAR-alpha-Striatal D1R Pathway to Reduce Fat Appetite in Obese Rats. *Cell Metab.* 2017;25(2):335-44.

2. Rakoff-Nahoum S, Paglino J, Eslami-Varzaneh F, Edberg S, and Medzhitov R. Recognition of commensal microflora by toll-like receptors is required for intestinal homeostasis. *Cell.* 2004;118(2):229-41.

3. Thaiss CA, Itav S, Rothschild D, Meijer MT, Levy M, Moresi C, et al. Persistent microbiome alterations modulate the rate of post-dieting weight regain. *Nature.* 2016;540(7634):544-51.

4. Thaiss CA, Itav S, Rothschild D, Meijer M, Levy M, Moresi C, et al. Persistent microbiome alterations modulate the rate of post-dieting weight regain. *Nature.* 2016.

5. Chevalier C, Stojanovic O, Colin DJ, Suarez-Zamorano N, Tarallo V, Veyrat-Durebex C, et al. Gut Microbiota Orchestrates Energy Homeostasis during Cold. *Cell.* 2015;163(6):1360-74.

6. Weitkunat K, Schumann S, Petzke KJ, Blaut M, Loh G, and Klaus S. Effects of dietary inulin on bacterial growth, short-chain fatty acid production and hepatic lipid metabolism in gnotobiotic mice. *J Nutr Biochem.* 2015;26(9):929-37.

7. Han J, Lin K, Sequeira C, and Borchers CH. An isotope-labeled chemical derivatization method for the quantitation of short-chain fatty acids in human feces by liquid chromatography-tandem mass spectrometry. *Anal Chim Acta.* 2015;854:86-94.

8. Haange SB, Jehmlich N, Hoffmann M, Weber K, Lehmann J, von Bergen M, et al. Disease Development Is Accompanied by Changes in Bacterial Protein Abundance and Functions in a Refined Model of Dextran Sulfate Sodium (DSS)-Induced Colitis. *J Proteome Res.* 2019;18(4):1774-86.

9. Haange S-B, Jehmlich N, Krügel U, Hintschich C, Wehrmann D, Hankir M, et al. Gastric bypass surgery in a rat model alters the community structure and functional composition of the intestinal microbiota independently of weight loss. *Microbiome.* 2020;8(1):13.

10. Kanehisa M, and Goto S. KEGG: kyoto encyclopedia of genes and genomes. *Nucleic Acids Res.* 2000;28(1):27-30.

11. Ihaka R, and Gentleman R. R: A Language for Data Analysis and Graphics. *Journal of Computational and Graphical Statistics.* 1996;5(3):299-314.

12. Dixon P. VEGAN, a package of R functions for community ecology. *Journal of Vegetation Science.* 2003;14(6):927-30.

13. Benjamini Y, and Hochberg Y. Controlling the False Discovery Rate - a Practical and Powerful Approach to Multiple Testing. *J Roy Stat Soc B Met.* 1995;57(1):289-300.

14. Wickham H. ggplot2. *Wiley Interdisciplinary Reviews: Computational Statistics.* 2011;3(2):180-5.


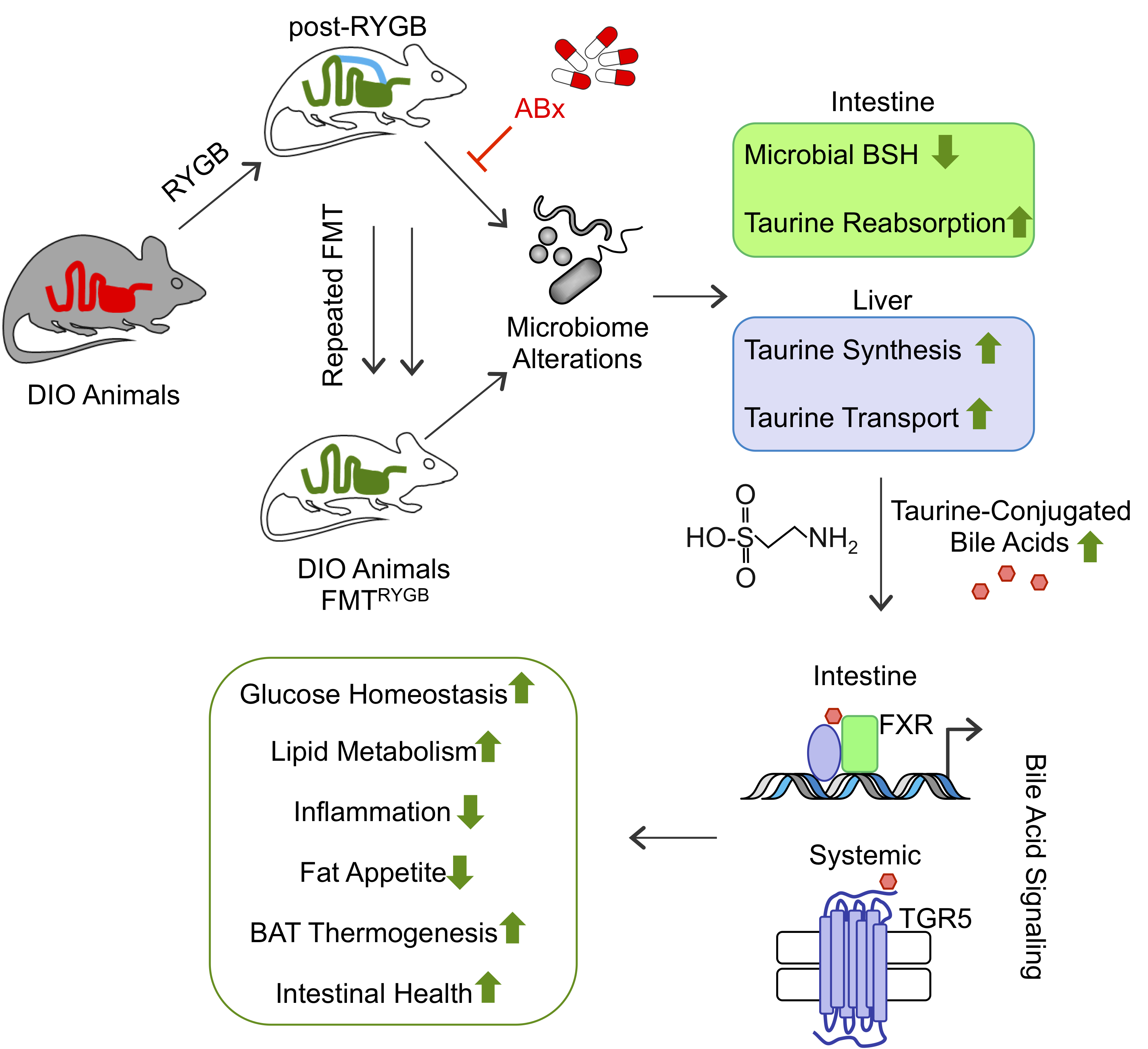


**Graphical Abstract.** Bariatric Surgery (i.e. RYGB) or the repeated fecal microbiota transfer (FMT) from RYGB donors into DIO (diet-induced obesity) animals induces shifts in the intestinal microbiome, an effect that can be impaired by oral application of antibiotics (ABx). Our current study shows that RYGB-dependent alterations in the intestinal microbiome result in an increase in the luminal and systemic pool of Taurine-conjugated Bile acids (TCBAs) by various cellular mechanisms acting in the intestine and the liver. TCBAs induce signaling via two different receptors, farnesoid X receptor (FXR, specifically in the intestines) and the G-protein-coupled bile acid receptor TGR5 (systemically), finally resulting in metabolic improvement and advanced weight management. BSH, bile salt hydrolase; BAT brown adipose tissue.

**Supplemental Figures**

**Supplemental Figure 1. Schematic of experimental design.** Top row illustrates experimental design of the ‘RYGB group’: Animals were fed with high fat diet (HFD) for 5 weeks, followed by RYGB surgery and a 2-week post-surgery recovery. For the following 5 weeks, animals received a two-choice diet with HFD and SC *ad libitum*. These animals were used as RYGB donors for the ‘FMT^RYGB^ group’. Middle row represents experimental design of the ‘RYGB(ABx) group’: Following the RYGB surgery and recovery phase, animals were placed on a two-choice diet with HFD and SC *ad libitum* and received orally administered antibiotics (ABx) for a time period of 5 weeks (see *Material and Methods* for composition of the antibiotic cocktail). Bottom row presents the schematic overview of the ‘FMT^RYGB^ group’: Animals were fed with a HFD for 7 weeks before diet was switched to a two-choice-diet with HFD and SC *ad libitum*. From then on, animals received weekly fecal microbiota transplantation (FMT) with fresh stool from RYGB donors for a time period of 5 weeks.

**Supplemental Figure 2. Lean microbiota depletion has no effect on host energy and glucose control. (a-d)** Relative body weight change (in %; øBW per group: LEAN W0: 475.1g, W5: 524.9g; LEAN(ABx) W0: 450.6g; W5: 489.3g) **(a)**, cumulative energy intake (in g) **(b)**, food efficiency (in %) **(c)**, energy excretion (in kJ/24 hours) **(d)** and dietary energy assimilation (in kJ/24 hours) **(e)** in SC-fed rats with 35-day antibiotic-treatment (ABx) compared to their respective controls (SC-fed rats without ABx). **(f-h)** Oral glucose-tolerance tests (oGTT) **(f)**, insulin-tolerance tests (ITT) **(g)**, and weight of BAT relative to body weight **(h)**.

**Supplemental Figure 3. Antibiotics treatment affects the host’s fecal microbiota abundance, food preference and mitochondrial respiration.** The overall bacterial load in animal feces is decreased 5 weeks after ABx treatment as compared to control animals without ABx (n=3 animals per group, measured in triplicate, bacterial DNA content normalized to feces weight, logarithmic scale, p=0.1022) **(a)**. Preference for High Fat Diet (HFD) increases significantly in RYGB animals after ABx treatment (RYGB(ABx)) as compared to non-ABx animals **(b)**. RYGB surgery increases parameters of mitochondrial respiration (basal Oxygen Consumption Rate (OCR) **(c)**, maximum respiration **(d)**, effect of norepinephrine **(e)**, in brown adipocytes treated with rat serum (or PBS as control). These effects are significantly diminished by ABx treatment. Data are mean ± s.e.m; n= 3-8 animals per group with pooled data from 2-3 independent experiments. Statistical analysis was performed with unpaired two-tailed Student’s *t*-test (for two groups) or two-way ANOVA (for multiple comparisons).

**Supplemental Figure 4. Lean microbiota transfer has no effect on host energy and glucose control in HFD-induced obesity. (a-d)** Relative body weight change (in %; øBW per group: FMT^LEAN^ W0: 504.2g, W5: 549.3g; DIO W0: 681.3g, W5: 762.3g) **(a)**, cumulative energy intake HFD *vs.* SC intake (in g) **(b)**, food efficiency (in %) **(c)**, energy excretion (in kJ/24 hours) **(d)** and dietary energy assimilation (in kJ/24 hours) **(e)** in HFD-induced obese (DIO) rats 5 weeks after fecal microbiota transfer (FMT) from SC-fed lean rats (FMT^LEAN^) compared to their respective controls (DIO rats not receiving FMT from lean animals). **(f-h)** Oral glucose-tolerance tests (oGTT) **(f)**, insulin-tolerance tests (ITT) **(g)**, and weight of BAT relative to body weight **(h)**. Data are mean ± s.e.m; n= 4-8 animals per group with pooled data from 2-3 independent experiments. Statistical analysis was performed with unpaired two-tailed Student’s t-test (for two groups) or two-way ANOVA (for multiple comparisons).

**Supplemental Figure 5. Reduced fat appetite is not the main factor for lower adiposity and improved host metabolism secondary to RYGB microbiota transfer into HFD-induced obesity. (a-c)** Cumulative HFD vs. SC intake (in g) **(a)**, relative body weight change (in %; øBW per group: PF(FMT^RYGB^) W0: 528.0g, W5: 595.2g; DIO W0: 681.3g, W5: 762.3g; FMT^RYGB^ W0: 588.8g, W5: 618.57g) **(b)** and adiposity index (fat mass/(fat mass + lean mass)) **(c)** in HFD-induced obese control rats (DIO), in DIO control rats which received RYGB fecal microbiota transfer (FMT^RYGB^) and in DIO rats which received the same amount of HFD and SC as consumed by FMT^RYGB^ rats (PF(FMT^RYGB^)). **(d-e)** BAT weight relative to body weight **(d)** and representative images of H&E stainings on BAT sections (scale bars 200 µm) **(e)**. **(f-h)** Oral glucose-tolerance tests (oGTT) **(f)**, insulin-tolerance tests (ITT) **(g)**, and OilRedO staining on sections from liver (scale bars 200 µm) **(h)**. Note that for clarity purposes and in order to reduce animal numbers, data from groups DIO and FMT^RYGB^ from main figure 2 have exceptionally been included as control groups in this supplementary figure. Data are mean ± s.e.m; n= 4-8 animals per group with pooled data from 2-3 independent experiments. **P* <0.05, ***P* <0.01, ****P* <0.001 as FMT^RYGB^ group compared with PF(FMT^RYGB^); ^#^*P* <0.05, ^##^*P* <0.01, ^###^*P* <0.001 as FMT^RYGB^ group compared to DIO control. Statistical analysis was performed with two-way ANOVA with Tukey correction for multiple testing.

**Supplemental Figure 6. Post-RYGB gut microbiota reduces adipose tissue fibrosis. (a, b)** Quantification of adipocyte cell size distribution (in %) in eWAT **(a)** and iWAT **(b)** fat depots of HFD-induced obese (DIO) rats 5 weeks after fecal microbiota transfer (FMT) from RYGB-operated rats (FMT^RYGB^) compared to their respective controls (DIO rats not receiving RYGB FMT). **(c-e)** Representative images of Sirius-red staining on sections from eWAT and iWAT fat depots (scale bars 200 µm) **(c)** with corresponding quantification of collagen deposition **(d)**. Representative images of Sirius-red staining on sections from BAT (scale bars 200 µm) and corresponding tissue fibrosis quantification **(e)**. Mitochondrial respiration of adipocytes is increased after incubation with serum from FMT(RYGB) rats as compared to control cells (PBS treatment) and cells treated with DIO serum, as shown for basal OCR **(f)**, maximal OCR **(g)**  and the inducing effect of epinephrine (NE) **(h)**. Data are mean ± s.e.m; n= 3-8 animals per group with pooled data from 2-3 independent experiments. ** *P*<0.01, ****P* <0.001; by unpaired two-tailed Student’s t-test.

**Supplemental Figure 7. Post-RYGB gut microbiota alters intestinal metabolites in HFD-induced obesity. (a-e)** Heatmap of clustering of cecal metabolites **(a)** in HFD-induced obese rats assigned to RYGB surgery (RYGB, with or without subsequent ABx, RYGB(ABx)), to transfer of post-RYGB fecal microbiota (FMT^RYGB^) or to no treatment (DIO). Weight (in g) and diameter (in cm) **(b)** of caeci. Total bile acid concentration (in nM/mg) **(c)**, primary **(d)** and secondary **(e)** bile acid concentration (in nM/mg) in caecum contents. **(f-k)** Relative and total amounts of unconjugated **(f, g)**, taurine (T)-conjugated **(h, i)** and glycine (G)-conjugated bile acids **(j, k)** (in nM/mg) in caecum contents. **(l-n)** Ratio of conjugated to unconjugated bile acids **(l)**, free taurine **(m)** and glycine **(n)** concentrations (in µM/mg) in caecum content. Data are mean ± s.e.m; n= 6-10 animals per group with pooled data from 3 independent experiments. * p<0.05, ** p<0.01, *** p<0.001; by unpaired Student’s t-test.

**Supplemental Figure 8. Post-RYGB gut microbiota improves small intestinal health by reducing inflammation, permeability and apoptosis. (a-d)** Relative mRNA gene expression of gut hormones in ileum **(a)**, representative images of H&E and PAS staining of ileum sections (scale bars 200 µm) **(b)** and corresponding morphometric quantification of the ileal vili length (in µm) **(c)** and goblet cell count per crypt **(d)** of HFD-induced obese (DIO) rats 5 weeks after fecal microbiota transfer (FMT) from RYGB-operated rats (FMT^RYGB^) compared to their respective controls (DIO rats not receiving RYGB FMT). **(e-i)** Relative mRNA expression of inflammatory cytokines **(e)** and mucosal defense genes **(f)** inileum, and intestinal permeability **(g, h)**. Assessment of apoptotic cells in ileal villi by terminal deoxynucleotidyl transferase (dUTP) nick and labeling (TUNEL) assay double-labeled with DAPI (scale bars 100 μm) **(i)**. Data are mean ± s.e.m; n= 5-8 animals per group with pooled data from 2-3 independent experiments. *p<0.05, **p<0.01, ***p<0.001; by unpaired Student’s t-test.

**Supplemental Figure 9. Gly-MCA *per se* shows no effect on energy and glucose control in HFD-induced obesity. (a, b)** Relative body weight change (in %; øBW per group: DIO(Gly-MCA) W0: 29.2g, W5: 33.0g; DIO W0: 29.1g; W5: 31.8g) under Gly-MCA treatment **(a)** and cumulative energy intake (in kcal) **(b)** of HFD-fed mice receiving 5 weeks treatment with Gly-MCA (DIO(Gly-MCA)) *versus* HFD-fed mice receiving no treatment with Gly-MCA (DIO control). **(c-f)** Oral glucose-tolerance tests (oGTT) **(c)**, insulin-sensitivity tests (ITT) **(d)**, liver weight relative to body weight **(e)**, and OilRedO staining of sections from liver (scale bars 100 µm) **(f)**. Data are mean ± s.e.m; n= 6-10 animals per group with pooled data from 3 independent experiments. Statistical analysis was performed with unpaired two-tailed Student’s *t*-test.

**Supplemental Figure 10. Gly-MCA does not affect hepatic FXR signaling but activates WAT browning. (a-c)** Intestine-specific FXR-inhibitor Gly-MCA shows no effect on FXR signaling in liver **(a)**. Liver weight relative to body weight **(b)** and cumulative energy intake (in g) **(c)** of HFD-induced obese (DIO) rats 5 weeks after fecal microbiota transfer (FMT) from RYGB-operated rats (FMT^RYGB^) compared to their respective controls (DIO rats not receiving RYGB FMT). **(d, e)** Representative images of H&E and UCP1 staining in iWAT (scale bars 100 µm) **(d)** and corresponding quantification of adipocyte cell size of iWAT (in %) **(e)**. Data are mean ± s.e.m; n= 6-10 animals per group with pooled data from 3 independent experiments. * p<0.05, ** p<0.01, *** p<0.001; statistical analysis was performed with unpaired two-tailed Student’s *t*-test.

**Supplemental Figure 11. RYGB microbiota transfer (FMT) largely fails to counter adiposity and affect metabolism in TGR5^-/-^ knockout mice.** **(a-b)** TGR5^-/-^ mice under FMT show only minor effects on body weight, here relative body weight change (in %; øBW per group: TGR5^-/-^(DIO) W0: 34.8g, W5: 39.8g; TGR5^-/-^(FMT) W5: 37.3g, W5: 41.6g) **(a)** despite unchanged appetite, here cumulative energy intake (in kcal) **(b)**. **(c-d)** Oral glucose-tolerance tests (oGTT) **(c)** and insulin-sensitivity tests (ITT) **(d)**. **(e-f)** Body temperature (in °C) under 4 hours of cold-exposure **(e)** and infrared images after 4 hours of cold-exposure **(f)**, indirect calorimetry results with heat production (in kcal/h) **(g)**, Oxygen consumption (VO_2_; in ml/h/kg) **(h)** and carbon dioxide production (VCO_2_; in ml/h/kg) **(i)** in TGR5^-/-^ mice with and without FMT from RYGB-operated animals. Data are mean ± s.e.m; n= 3-10 animals per group with pooled data from 2-3 independent experiments. * p<0.05, ** p<0.01, *** p<0.001; statistical analysis was performed with unpaired two-tailed Student’s *t*-test.

**Supplementary Tables**

**Supplementary Table S1. Pathway analysis.** The significance of changes in relative pathway abundance was determined by ANOVA with the P value corrected for multiple testing (Benjamini-Hochberg) followed by a Tukey posthoc test to determine significant differences between group pairings.
